# Gut dysbiosis following organophosphate, diisopropylfluorophosphate (DFP), intoxication and saracatinib oral administration

**DOI:** 10.3389/frmbi.2022.1006078

**Published:** 2022-10-20

**Authors:** Meghan Gage, Akhil A. Vinithakumari, Shankumar Mooyottu, Thimmasettappa Thippeswamy

**Affiliations:** 1 Interdepartmental Neuroscience, The Departments of Biomedical Sciences, Iowa State University, Ames, IA, United States; 2 Veterinary Pathology, College of Veterinary Medicine, Iowa State University, Ames, IA, United States

**Keywords:** organophosphate nerve agent, diisopropylfluorophosphate (DFP), gut microbiome, saracatinib, orally administered drugs

## Abstract

Organophosphate nerve agents (OPNAs) act as irreversible inhibitors of acetylcholinesterase and can lead to cholinergic crisis including salivation, lacrimation, urination, defecation, gastrointestinal distress, respiratory distress, and seizures. Although the OPNAs have been studied in the past few decades, little is known about the impact on the gut microbiome which has become of increasing interest across fields. In this study, we challenged animals with the OPNA, diisopropylfluorophosphate (DFP, 4mg/kg, s.c.) followed immediately by 2mg/kg atropine sulfate (i.m.) and 25mg/kg 2-pralidoxime (i.m.) and 30 minutes later by 3mg/kg midazolam (i.m.). One hour after midazolam, animals were treated with a dosing regimen of saracatinib (SAR, 20mg/kg, oral), a src family kinase inhibitor, to mitigate DFP-induced neurotoxicity. We collected fecal samples 48 hours, 7 days, and 5 weeks post DFP intoxication. 16S rRNA genes (V4) were amplified to identify the bacterial composition. At 48 hours, a significant increase in the abundance of *Proteobacteria* and decrease in the abundance of *Firmicutes* were observed in DFP treated animals. At 7 days there was a significant reduction in *Firmicutes* and *Actinobacteria*, but a significant increase in *Bacteroidetes* in the DFP groups compared to controls. The taxonomic changes at 5 weeks were negligible. There was no impact of SAR administration on microbial composition. There was a significant DFP-induced reduction in alpha diversity at 48 hours but not at 7 days and 5 weeks. There appeared to be an impact of DFP on beta diversity at 48 hours and 7 days but not at 5 weeks. In conclusion, acute doses of DFP lead to short-term gut dysbiosis and SAR had no effect. Understanding the role of gut dysbiosis in long-term toxicity may reveal therapeutic targets.

## 1 Introduction

Organophosphate nerve agents (OPNAs) have been utilized in the past to target both military and civilian populations (Morita et al., 1995; Tucker, 1996; Okumura et al., 1998; Yanagisawa et al., 2006). These nerve agents, including soman, sarin, cyclosarin, VX, tabun, and several others, are irreversible inhibitors of acetylcholinesterase (Mukherjee and Gupta, 2020). One-time exposure to a subacute dose of OPNA can lead to cholinergic crisis which includes symptoms such as salivation, lacrimation, urination, pupillary constriction, gastrointestinal distress, bronchoconstriction, muscle weakness and convulsions (Jett, 2012). In higher doses of OPNA acute exposure, the convulsions can lead to the development of *status epilepticus* (SE) which in turn, initiates epileptogenesis and the development of spontaneously recurring seizures (Jett, 2007; Rojas et al., 2018; Putra et al., 2020c). Currently, medical countermeasures for the short-term effects of OPNA toxicity include cholinergic receptor antagonists (atropine), acetylcholinesterase reactivators (HI-6, 2-pralidoxime (2-PAM)), and GABAergic agonists (benzodiazepines). Although there are many anti-seizure drugs available to aide in the long-term neurological consequences of OPNA toxicity, about 30% of people with epilepsy develop pharmacoresistance and do not respond to current medications (French, 2007). This highlights the need to find new therapeutic avenues.

Although our understanding of the neurological consequences of OPNA-induced neurotoxicity has been of great interest in recent decades, less is known about other short- and long-term toxicity in other biological systems including the gut microbiome. One study, showed that there was a significant effect on the fecal bacteria biota and urine metabolites following soman intoxication (Getnet et al., 2018). Until this study, this was an unknown consequence of OPNA toxicity and has not been studied in other models of OPNA intoxication. In the current study, we investigated the changes in the fecal microbiome following intoxication with the OPNA, diisopropylfluorophosphate (DFP). DFP, though less potent than other OPNAs used in real-world chemical warfare scenarios is often used as a surrogate to model the effects of OPNAs (Lim et al., 1983; Deshpande et al., 2010; Flannery et al., 2016; Puttachary et al., 2016).

In addition to determining the effects of DFP on the fecal microbiome, we also analyzed the fecal microbiome from animals that were administered the potential disease modifier, saracatinib (SAR) *via* the oral route. SAR is a potent inhibitor of src tyrosine family kinases (SFKs) which are involved in a variety of biological processes (Green et al., 2009; Nie et al., 2020). SAR has been previously used in both preclinical and clinical studies of cancer, Alzheimer’s disease (AD), and epilepsy (Baselga et al., 2010; Kaufman et al., 2015; Sharma et al., 2018; Smith et al., 2018; Sharma et al., 2021; Luo et al., 2021). We have previously shown that SAR treatment when given soon after DFP intoxication, reduces seizures, some behavioral deficits, as well as both short- and long-term gliosis and neurodegeneration (Gage et al., 2021a; Gage et al., 2021b). Having known the potential disease-modifying effects of orally administered SAR in experimental models, it is important to understand the impact of SAR alone, on the microbiome, as well the impact of SAR in the context of OPNA intoxication from therapeutic perspective.

## 2 Materials and methods

### 2.1 Animals, care and ethics

Male Sprague Dawley rats (7-8 weeks) were purchased from Charles River (Wilmington, MA, USA). The fecal samples (n=6/time-point) for this study were from the same animals that were reported in our recent publications on the disease-modifying effects of SAR in the rat DFP model (24, 25). The data on the gut microbiota presented here are novel. Though no post-mortem data is presented here, at the end of experiment, all animals used in this study were euthanized with 100mg/kg pentobarbital sodium (Iowa State University Llyod Veterinary Medical Center Hospital Pharmacy). Procedures were approved by the Iowa State Institutional Care and Use Committee (IACUC-18-159). Animals were single housed at the Iowa State Laboratory of Animal resources, given *ab libitum* access to food and water and light/dark cycles of 12 hours. All procedures were complied with the ARRIVE guidelines (Kilkenny et al., 2010).

### 2.2 DFP intoxication

**FIGURE 1:**
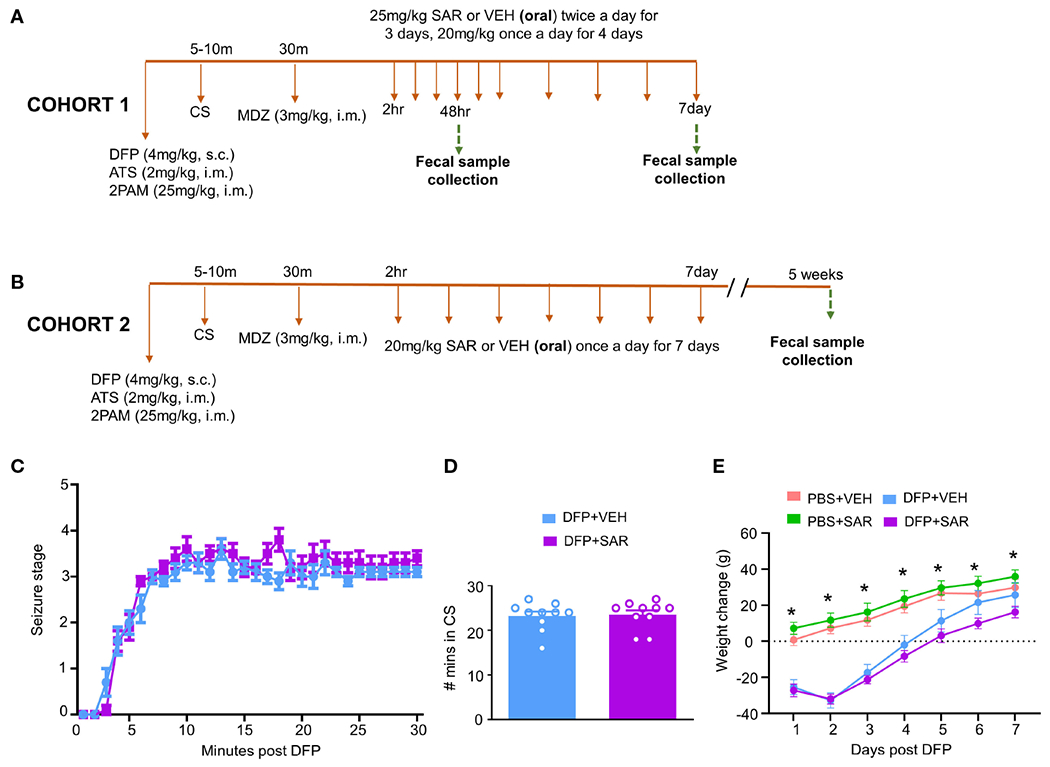
Experimental design and impact of DFP and SAR on seizures and bodyweight changes. **(A, B)** Two cohorts of animals were used in this experiment. Both cohorts were challenged with 4mg/kg diisopropylfluorophosphate (DFP, s.c.) followed immediately by 2mg/kg atropine sulfate (ATS, i.m.) and 25mg/kg 2-pralidoxime (2-PAM, i.m.) and one hour later 3mg/kg midazolam (MDZ, i.m.). Two hours after MDZ, saracatinib (SAR) or vehicle (VEH) treatment began. Cohort-1 received 25mg/kg twice a day for the first three days followed by 20mg/kg once a day for four days with fecal collections at 48 hours and 7 days. The second cohort received seven daily doses 20mg/kg SAR and had fecal collections at 5 weeks. **(C)** Seizure response over time, linear mixed effects model. **(D)** Number of minutes animals spent in a convulsive seizure (CS), t-test. **(E)** Bodyweight changes over the treatment period. Linear mixed effects model, *p < 0.05.

The experimental design is outlined in Figures 1A, B. Animals were exposed to 4 mg/kg DFP (s.c.) followed immediately by 2 mg/kg atropine sulfate (ATS, i.m.) and 25 mg/kg 2-PAM (i.m.) to reduce mortality and to counteract the peripheral effects of AChE inhibition. Control animals were administered phosphate buffered saline (PBS). To confirm the toxicity caused by DFP, animals were assessed for seizure score based on a modified Racine sale as described in our previous publications (Racine, 1972; Putra et al., 2020a; Gage et al., 2021a). Stages 1 (salivation, lacrimation, urination, defecation-SLUD), and mastication, and 2 (head nodding, tremors) were considered nonconvulsive seizures (NCS). Stages 3 (rearing, Staub tail, forelimb clonus), 4 (falling, loss of righting reflex), and 5 (repeated falling, abducted limbs, rapid circling) were considered convulsive seizures (CS). To control behavioral seizures, animals were administered 3 mg/kg midazolam (MDZ, i.m.) once the animals spent 20 minutes in CS (approximately 30 minutes after DFP). SAR or vehicle (VEH) was administered orally 2 hours after MDZ. VEH consisted of 0.5% hydroxypropyl methylcellulose and 0.1% tween 20; preparation as described in our previous publication (Gage et al., 2021a). SAR was left stirring during the experiment to avoid precipitation. Importantly, DFP treated animals were randomly assigned to either SAR or VEH treatment so that the groups had equal SE severity.

Two cohorts of animals were used in this study. In the first cohort, 25 mg/kg SAR or vehicle was administered twice a day for the first 3 days followed by 20 mg/kg once a day for four days. Fecal samples were collected from these animals 48 hours and 7 days post DFP intoxication. In order to understand the long-term impact of DFP and SAR on the microbiome, we collected fecal samples from a second cohort of animals 5 weeks post DFP from animals in another study which has already been published (Gage et al., 2021b) except the microbiome data. In this group, 20 mg/kg SAR or VEH was administered once a day for 7 days to understand the impact of lower doses of SAR effect on the gut microbiome in the long term

### 2.3 Fecal sample collection and sequencing

Fresh samples were collected from each animal 48 hours, 7 days and 5 weeks after DFP intoxication. Extraction and sequencing have been previously described (Mooyottu et al., 2017). The DNA for 16S rRNA gene sequencing was isolated from 10 to 50 mg fecal pellet from each rat using the DNeasy PowerSoil HTP 96 Kit (Qiagen, Valencia, CA, USA, Cat# 12955-4) according to the manufacturer’s instructions. The 16S rRNA sequencing library was created as previously reported (Kozich et al., 2013). The hypervariable V4 region of the 16S rRNA gene was then targeted using PCR with the forward primer 515F (5′GTGCCAGCMGCCGCGGTAA3′) and the reverse primer 806R (5′GGACTACNNGGGTATCTAAT3′). The 16S rRNA amplicon library was sequenced using the MiSeq technology (Illumina, San Diego, CA). The Sequal Prep normalization plate kit was used to normalize the cleaned amplicons (Thermo Fisher Scientific, Waltham, CA). A sequencing library was built according to the manufacturer’s methodology, and sequencing was performed using the Illumina Hiseq 2500 platform.

### 2.4 Data analysis

Qiime 2 (v2021.11) was used for all sequence processing steps (Bolyen et al., 2019). Noisy sequencing data were removed, including error tags, chimera, and low-quality sequences using cutadapt (Martin, 2011). At 97% identity, the clean data were grouped into operational taxonomic units (OTUs) and compared to Greengenes databases (Release 13.8). Comprehensive differential abundance analyses were performed using the MicrobiomeAnalyst web-based platform (https://www.microbiomeanalyst.ca/) according to the relative abundance of OTUs (Chong et al., 2020). Marker Data Profiling was used to examine gene abundance data (MDP). Data were rarified to a minimum library size and filtered to exclude features with less than four counts and less than 20% prevalence. The alpha and beta diversity data were calculated with the MicrobiomeAnalyst platform according to the relative abundance of OTUs. The linear discriminant analysis (LDA) effect size (LEfSe) was also used to identify the various taxonomies.

### 2.5 Statistical analysis

Graphpad prism 9.3.0 software and MicrobiomeAnalyst were used to analyze and graph the results. Linear analyses were primarily used to analyze the significance of results. Sharpiro-Wilcox tests were used to assess data normality. Experimenters were blind to treatment where appropriate and treatment groups were randomized with respect to SE severity.

## 3 Results

### 3.1 Initial response to DFP

The experimental designs are shown in Figures 1A, B. Animals developed CS within 5-10 minutes of DFP (4mg/kg) and were given MDZ about 30 minutes later so that animals had approximately 20 minutes of CS. There was no difference in SE severity between VEH-treated animals and SAR-treated animals over time (Figure 1C) or in the total number of minutes spent in a CS (Figure 1D). Both DFP treated groups lost weight for at least the first 2 days and began to steadily gain weight thereafter (Figure 1E). SAR administration did not impact the weight loss or weight gain (Figure 1E).

### 3.2 DFP and SAR short- and long-term impact on major gut microbiota

#### 3.2.1 48 hours post-DFP/SAR

**FIGURE 2:**
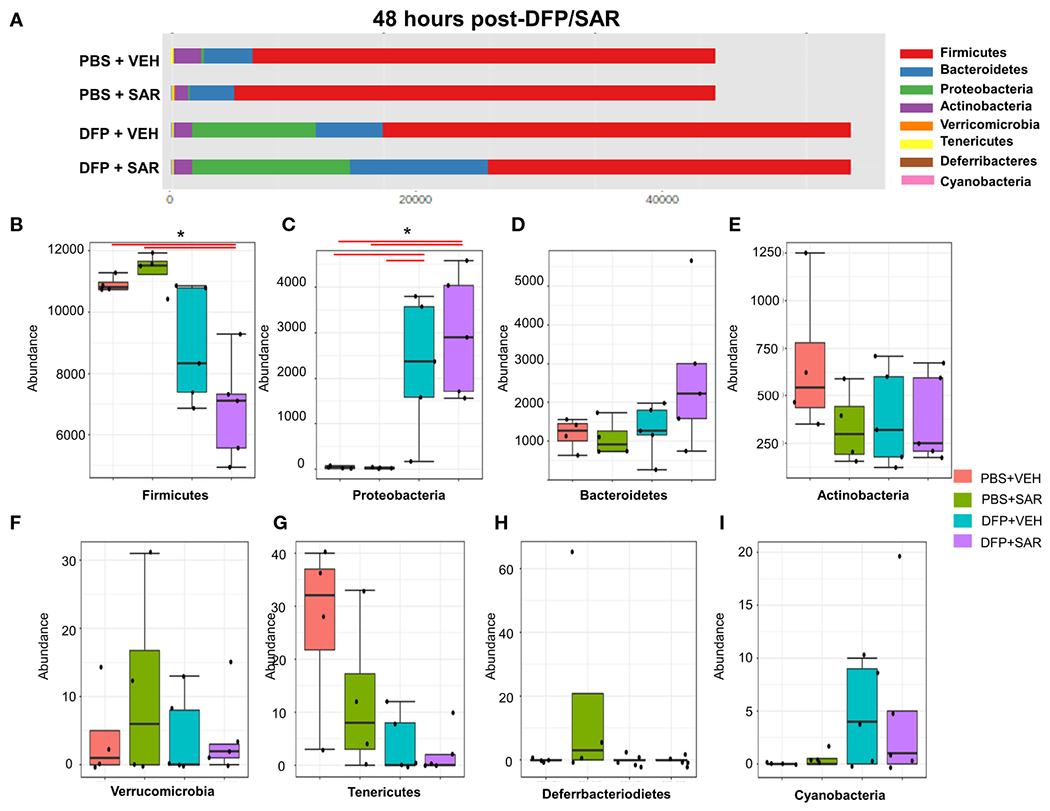
Impact of DFP and SAR on the phyla level at 48 hours post-exposure. **(A)** Overall changes and actual abundance in phyla. **(B–I)** Actual abundance by phyla for *Firmicutes* **(B)**, *Bacteroidetes* **(C)**, *Proteobacteria* **(D)**, *Actinobacteria* **(E)**, *Verricomicrobia* **(F)**, *Tenericutes* **(G)**, *Deferrbacteriodietes* **(H)**, and *Cynanobacteria* **(I)**. ANOVA or Kruskal Wallis test, *p<0.05, n=4-5.

**FIGURE 3:**
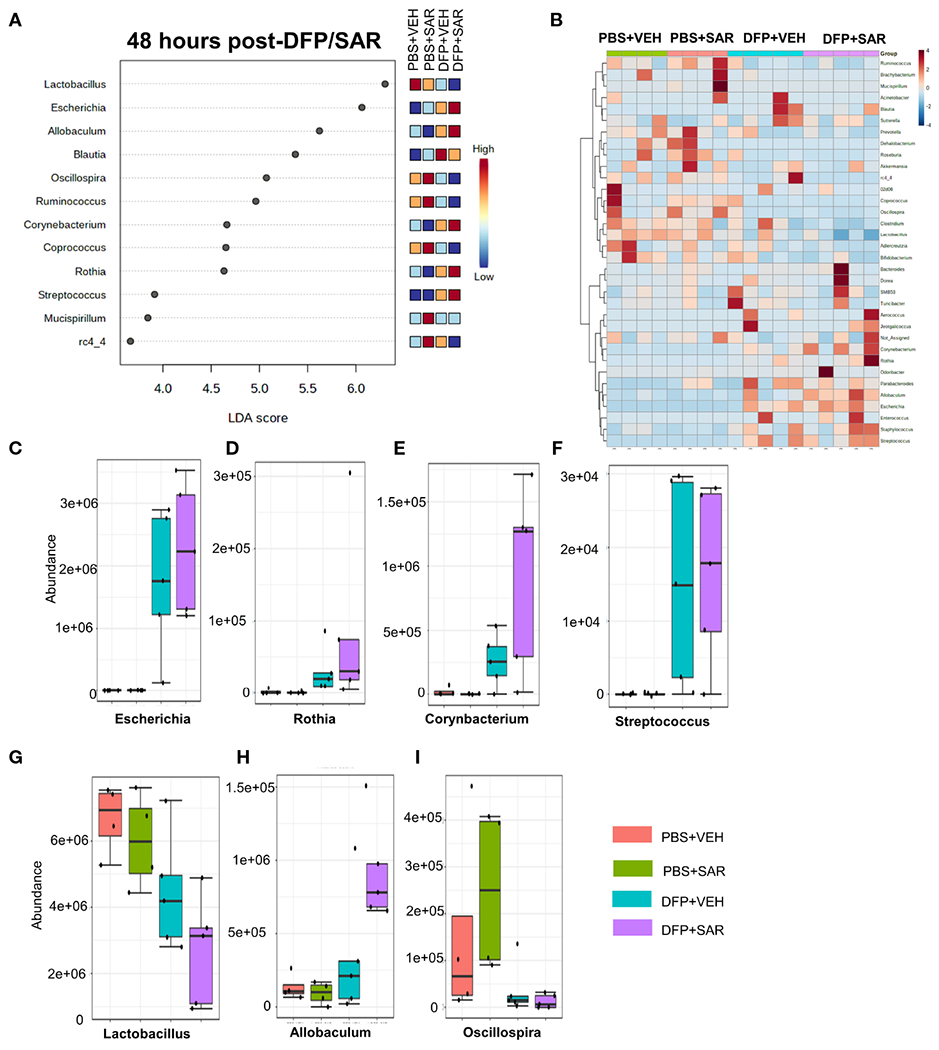
Impact of DFP and SAR on the genus level at 48 hours post-exposure. **(A)** LEfSe analysis revealed genera with LDA scores above 2.0. **(B)** Heatmap clustering by genus. **(C-I)**. Trends in actual abundance of the seven genera with an overall p<0.05, n=6.

Univariate analysis was performed by MicrobiomeAnalyst software to determine the impact of treatment on phyla at each timepoint. The overall impact of DFP and SAR on gut microbiota phyla at 48 hours is shown in Figure 2A. Class through species levels are represented in Figure S1. The abundance for each phylum is presented in Figures 2B–I. There was a DFP-induced decrease in *Firmicutes* in both VEH and SAR treated groups, but it was only significant in the DFP +SAR group (Figure 2B). In contrast, there was a significant increase in *Proteobacteria* in both DFP treated groups compared to the controls (Figure 2C). However, there were no significant changes in *Bacteroidetes* (Figure 2D), *Actinobacteria* (Figure 2E), *Verrcomicrobia* (Figure 2F), *Tenericutes* (Figure 2G), *Deferrbacteriodetes* (Figure 2H) or *Cyanobacteria* (Figure 2I). Linear discriminant analysis (LDA) effect size (LEfSe) was used to observe group differences on the genus level. Genera with LDA scores above 2.0 are shown in Figure 3A. A heatmap cluster for the genera is presented in Figure 3B. Those with factor level p< 0.05 are represented by box plots in Figures 3C–I. In both DFP groups, there was an increase in *Escherichia, Rothia, Corynbacterium* and *Streptococcus* (Figures 3C–F). In the DFP +SAR group there was an increase in *Allobaculum* compared to all other groups (Figure 3H). There was DFP-induced reduction in *Lactobacillus*, and *Oscillospira* regardless of treatment with VEH or SAR (Figures 3G, I). Comparison of the treatment groups at 48 hours by species level is represented in Figure S2. The software, Picrust2, was used to predict the functional composition of the microbiota in the treatment groups; a heatmap is presented in Figure S3.

#### 3.2.2 7 days post-DFP/SAR

**FIGURE 4:**
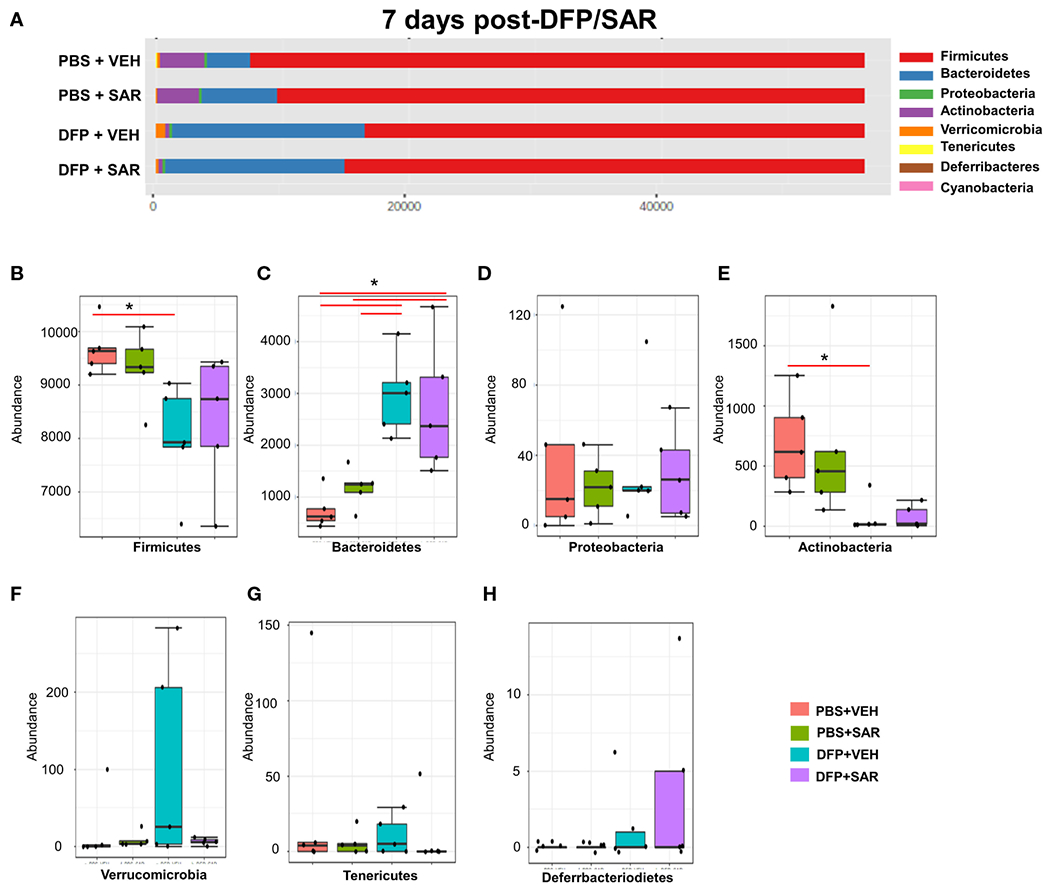
Impact of DFP and SAR on the phyla level at 7 days post-exposure. **(A)** Overall changes in actual abundance in phyla. **(B-I)** Actual abundance by phyla for *Firmicutes* **(B)**, *Bacteroidetes* **(C)**, *Proteobacteria* **(D)**, *Actinobacteria* **(E)**, *Verricomicrobia* **(F)**, *Tenericutes* **(G)**, *Deferrbacteriodietes* **(H)**. ANOVA or Kruskal Wallis test, *p < 0.05, n=4-5.

**FIGURE 5:**
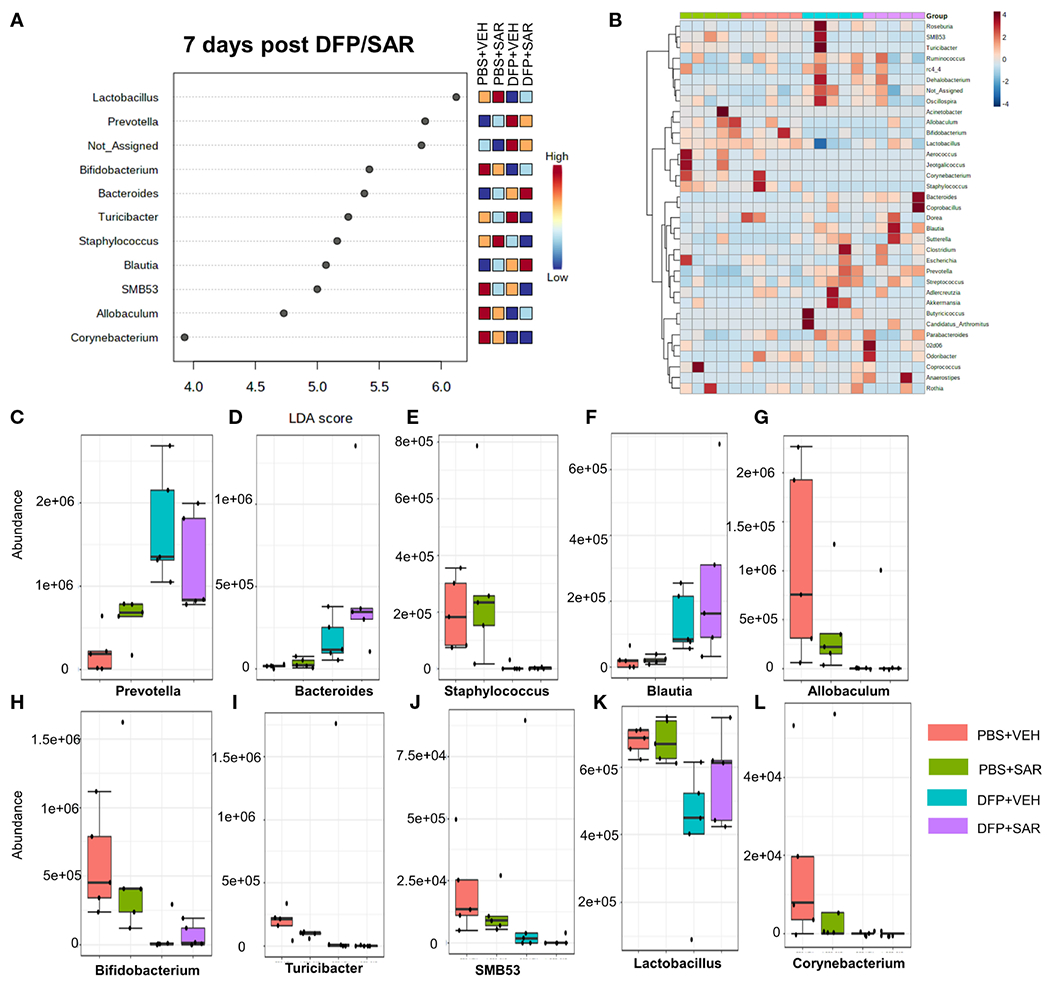
Impact of DFP and SAR on the genus level at 7 days post-exposure. **(A)** LEfSe analysis reveals genera with LDA scores above 2.0. **(B)** Heatmap clustering by genus. **(C–L)**. Trends in actual abundance of the seven genera with an overall p < 0.05, n=4-5.

The impact of DFP and SAR on gut microbiota at 7 days on the phyla level is shown in Figure 4A. Class to species levels are represented in Figure S4. There was a DFP-induced significant increase in *Bacteroidetes* (Figure 4C). Compared to the PBS +VEH control, the DFP+VEH animals had decreased *Firmicutes* and *Actinobacteria* (Figures 4B, E). There were no changes between the treatment groups on the other phyla levels (Figures 4D, F–H). Following LEfSe analysis, the genera with the highest LDA scores are presented in Figure 5A. A heatmap cluster by genus is presented in Figure 5B. Trends for genera with an overall p<0.05 are graphed in Figures 5C–L. In the DFP groups compared to the controls, there was an increased abundance of *Prevotella, Bacteroides*, and *Blautia* (Figures 5C, D, F) and a decrease in *Staphylococcus, Allobaculum, Bifidobacterium, Turicibacter, SMB53, Lactobacillus* and *Corynebacterium* (Figures 5E, G, H–L). Comparison of the treatment groups at 7 days by species level is represented in Figure S5.

#### 3.2.3 5 weeks post-DFP/SAR

**FIGURE 6:**
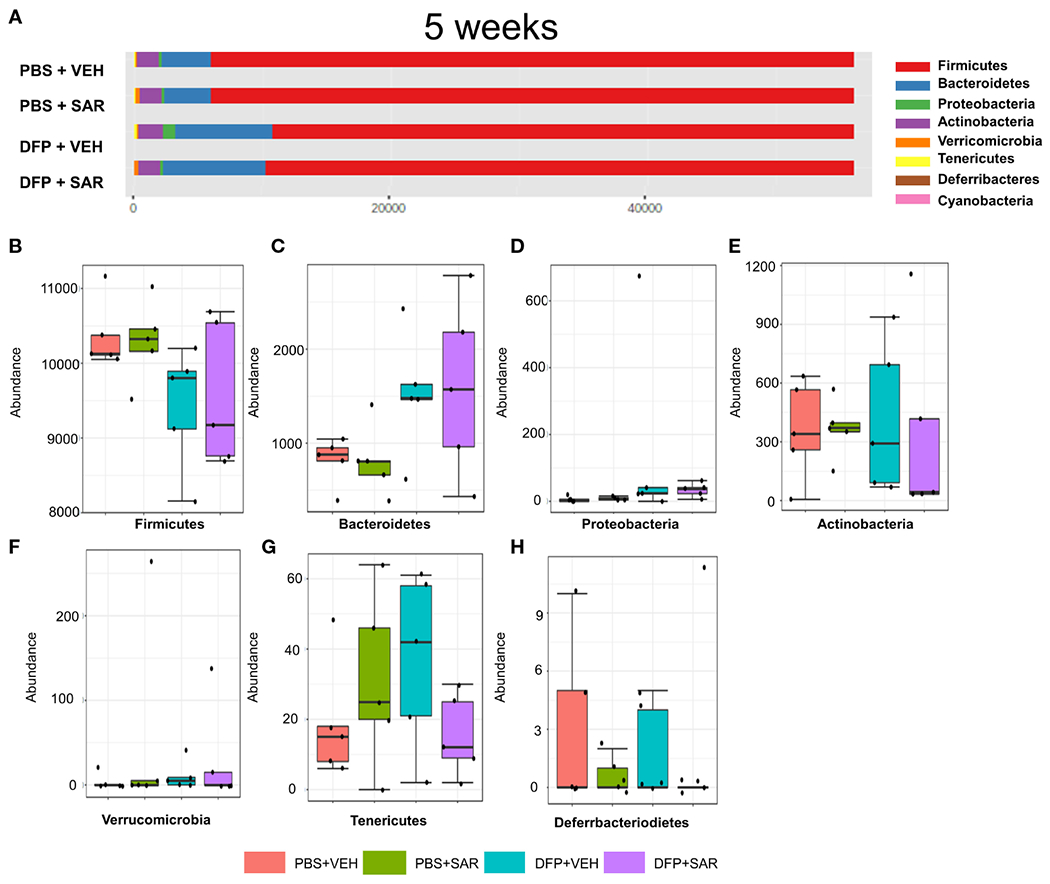
Impact of DFP and SAR on the phyla level at 5 weeks post-exposure. **(A)** Overall changes in phyla actual abundance. **(B–I)** Actual abundance by phyla for *Firmicutes* **(B)**, *Bacteroidetes* **(C)**, *Proteobacteria* **(D)**, *Actinobacteria* **(E)**, *Verricomicrobia* **(F)**, *Tenericutes* **(G)**, *Deferrbacteriodietes* **(H)**. ANOVA or Kruskal Wallis test, n=4-5.

**FIGURE 7:**
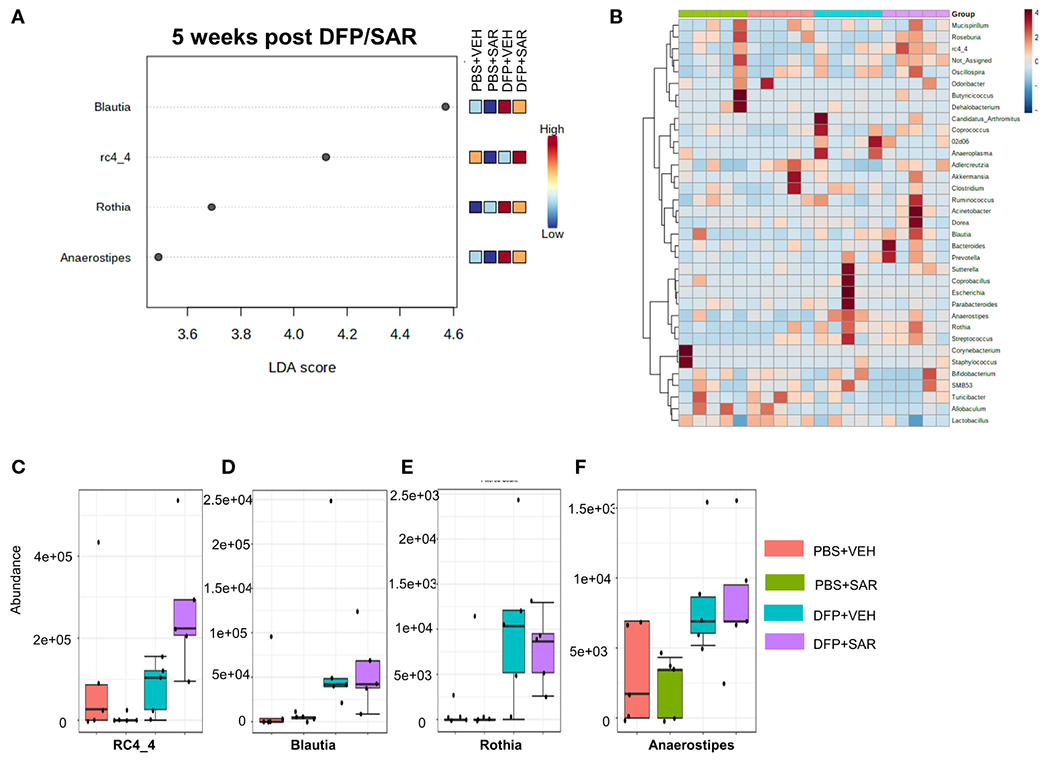
Impact of DFP and SAR on the genus level at 5 weeks post-exposure. **(A)** LEfSe analysis reveals genera with LDA scores above 2.0. **(B)** Heatmap clustering by genus. **(C–F)** Trends in actual abundance of the seven genera with an overall p-value<0.05, n=4-5.

The overall impact of DFP and SAR on gut microbiota phyla at 5 weeks is shown in Figure 6A. Order to species levels are represented in Figure S6. There were no significant differences between the treatment groups on any phyla level (Figures 6B–H). Following LEfSe analysis, the genera with the highest LDA scores are presented in Figure 7A. A heatmap by genus level is presented in Figure 7B. In the DFP groups compared to the controls, there was an increase in *RC4_4, Blautia, Rothia*, and *Anerostipes* (Figures 7C–F). Heat-trees at the species level are shown in Figure S7.

### 3.3 Impact of DFP and SAR on alpha and beta diversity at 48hrs, 7 days, and 5 weeks

**FIGURE 8:**
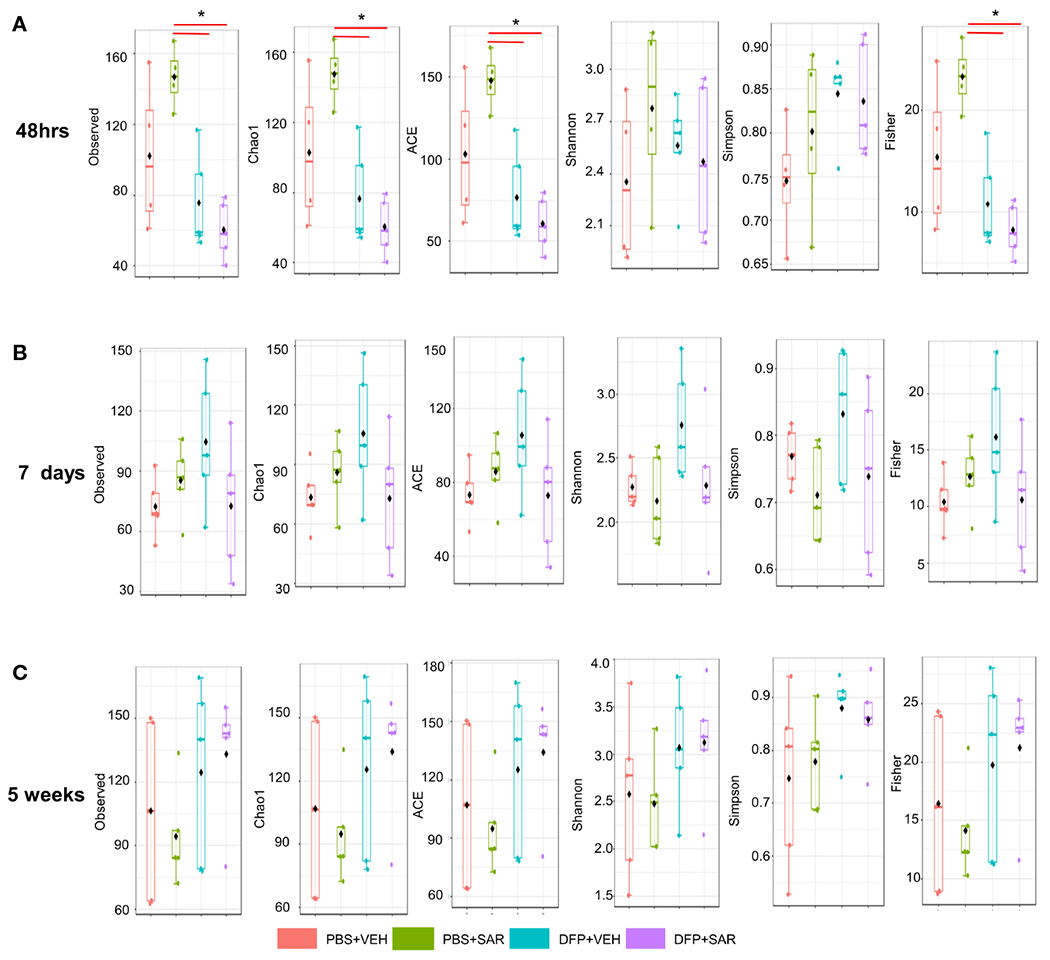
Alpha Diversity. Alpha diversity was measured using several metrics including observed, chao1, ACE, Shannon, Simpson, and Fisher at 48 hours **(A)**, 7 days **(B)** and 5 weeks **(C)** post-exposure, ANOVA or Kruskal Wallis test, *p < 0.05, n=4-5.

**FIGURE 9:**
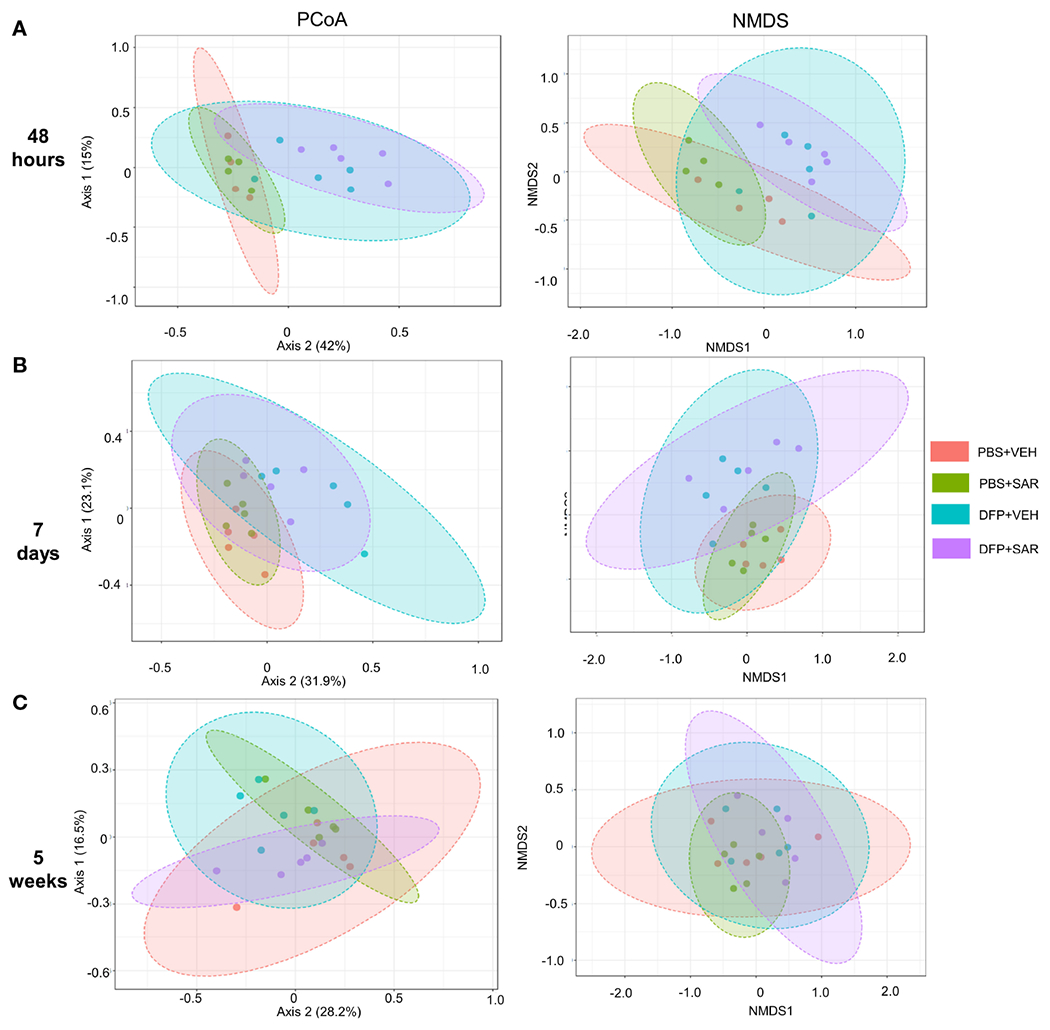
Beta diversity. Beta diversity was assed using principle coordinate analysis (PCoA) and multidimensional scaling (NMDS) at 48 hours **(A)**, 7 days **(B)** and 5 weeks **(C)** post-exposure.

We utilized observed (richness), chao1 and ACE (richness accounting for unobserved species), and Shannon, Simpson and Fisher (richness and evenness) to assess Alpha diversity among the treatment groups. At 48 hours, there was a significant reduction in observed, Chao1, ACE, and Fisher’s alpha diversity in the DFP-treated groups, both VEH and SAR, compared to the PBS+SAR treated group (Figure 8A). There was no significant difference between the treatment groups at 7 days or 5 weeks on any alpha diversity metric (Figures 8B, C). We measured beta diversity using a principal coordinate analysis (PCoA) and nonmetric multidimensional scaling (NMDS). The PERMANOVA results revealed that at all time points, treatment significantly contributed to the clustering (Figure 9). The PERMANOVA values are summarized in Table S1. The most dramatic clustering of samples was observed in the PCoA analysis. The DFP treated groups were clustered separately from the PBS treated groups at 48 hours and 7 days but not at 5 weeks (Figure 9).

## 4 Discussion

The purpose of the study was to determine the impact of the OPNA, DFP, and saracatinib on the gut microbiome. The SLUD and seizures response to DFP was similar to the previous studies from our lab and others (Rojas et al., 2018; Wu et al., 2018; Guignet et al., 2019; Putra et al., 2020a; Putra et al., 2020b; Gage et al., 2021a). In addition, we observed other DFP-induced changes such as exophthalmos, porphyrin staining, hunched body posture, tremors, muscle weakness, piloerection, and change in fur color in the days immediately following DFP intoxication (Irwin, 1968; Gage et al., 2021b). Animals developed CS 5-10 minutes post insult. Notably, we limited the duration of CS, i.e., SE, to about 20 minutes, similar to our previous studies (Gage et al., 2021a; Gage et al., 2021b). Although the seizure response to acute exposures to OPs and other OP-related consequences are well documented, the impact on the microbiome in not well known. In recent years, there has been increased interest in understanding the gut-brain axis and how neurological injury contributes to changes in the gut and vice versa (Carabotti et al., 2015; Panther et al., 2022).

Prior to this study, one other group did observe alterations in the gut microbiome following soman, an OPNA, intoxication (Getnet et al., 2018). DFP is typically used as a surrogate for soman or other OPNAs and has the same mechanism of action and clinical signs upon exposure (Jett, 2012). Soman. exposure increased in the relative abundance of *Proteobacteria* and *Cyanobacteria* 72 hours post exposure (Getnet et al., 2018). This was also observed in the current study, specifically at 48 hours post-DFP exposure. The mechanisms of DFP-induced gut dysbiosis are unclear. The gut microbiome has been primarily studied with respect to diet and obesity (Hills et al., 2019). Notably, DFP-exposed animals eat less in the 2-3 days following DFP intoxication, and therefore lose bodyweight. A number of bacteria have been found to contain genes implicated in metabolism of OPs, which could explain the increase in abundance of *Proteobacteria* and *Cyanobacteria* (Karpouzas and Singh, 2006; Getnet et al., 2018). Future studies could determine if any of the genera/species identified in this study are capable of OP metabolism. Interestingly, in the soman study (Getnet et al., 2018), the change in phyla was dependent upon the initial seizure response to soman which suggests that the gut dysbiosis is more dependent on the gut-brain axis rather than the administration of the organophosphate itself. In our study, all the animals had at least 20 minutes CS during SE, so we were not able to discriminate between the effects of seizures and the impact of DFP. Future studies could determine the impact of SE severity and duration on gut dysbiosis.

On a phyla level, it appears that in the short-term (48 hours), increase in *Proteobacteria* and decrease in *Firmicutes* are primarily responsible for gut dysbiosis. Increase in the abundance of *Proteobacteria* is considered to be a microbial signature of dysbiosis and is implicated in a wide variety of diseases, especially in those involving inflammation or metabolic dysfunction (Shin et al., 2015; Rizzatti et al., 2017). *Proteobacteria* are one of the most abundant phyla and are comprised of organisms with varying physiology (Shin et al., 2015). Upon analysis at the genus level, we found that the *Proteobacteria* increase in the DFP treated animals included *Escherichia*. Certain strains of *Escherichia* can act as an intestinal pathogen and could contribute to the poor health of the animals. In this study, in the days immediately following DFP intoxication there was the loss in bodyweight in the first 2-3 days post intoxication.

*Firmicutes* are thought to primarily be involved in fermenting short-chain fatty acids which impact the function of the intestinal barrier (Stojanov et al., 2020). The ratio of *Firmicutes* to *Bacteroidetes* has been implicated in several other diseases including obesity, inflammatory bowel syndrome, and major depressive disorder (Huang et al., 2018; Magne et al., 2020; Stojanov et al., 2020). In DFP-treated animals, there was a reduction in *Firmicutes* at 48 hours but an increase in the *Streptococcus* genera. There was a decrease in the *Lactobacillus* and *Oscillospira* genera, which have both been implicated as possible therapeutic targets *via* probiotics in other diseases (Di Cerbo et al., 2016; Yang et al., 2021). Possibly this phylum may be a therapeutic target too in OP intoxication as an adjunct therapy to mitigate DFP-induced gut dysbiosis.

Unlike the increase in *Proteobacteria*, the decrease in *Firmicutes* persisted at day 7 post-DFP. Interestingly, the LEfSe analysis revealed a reduction in *Staphylococcus* at 7 days in contrast to 48 hours post exposure. There was also a reduction in *Allobaculum, Lactobacillus, SMB53*, and *Turibacter*. There was also an increase in the *Bacteroidetes* phylum and reduction in *Actinobacteria. Bacteroidetes* are most well-known to be involved in the degradation of biopolymers in the intestine (Thomas et al., 2011). Based on the LEfSe analysis, the increase in *Bacteroidetes* seemed to be primarily driven by the increase in *Prevotella* and *Bacteroides* which are both implicated in health and disease (Su et al., 2018; Zafar and Saier, 2021). *Actinobacteria*, though less abundant than *Bacteroidetes* and *Firmicutes*, play an important role in gut homeostasis and are involved in a variety of processes including biotransformation, lipid and nutrient metabolism (Binda et al., 2018). It appears that the most highly affected *Actinobacteria* include *Bifidobacterium* and *Corynebacterim*. Like *Firmicutes, Actinobacteria* have also been the target of probiotics in disease (Binda et al., 2018).

Many of the changes we observed at 7 days were similar to those in previous studies of chronic exposure to less potent OPs such as those found in pesticides (Roman et al., 2019; Giambò et al., 2021). In a chlorpyrifos study, exposure during the gestational period led to alterations in the intestinal villi and changes in the microbiome composition with a reduction in *Lactobacillus* (Condette et al., 2015). Another chronic exposure study in mice, administered chlorpyrifos for 30 days and found a significant increase in the abundance of *Bacteroidetes* and significant decrease in the abundance of *Firmicutes* (Zhao et al., 2016). In an *in vitro* model of the human gut, an increase in *Bacteroides* and a reduction in *Bifidobacteria* have been reported (Reygner et al., 2016). Chronic oral administration of chlorpyrifos in various species led to gut microbiome changes in a diet dependent manner (Fang et al., 2018). Notably, most of these studies did not report the dramatic increase in *Proteobacteria* that observed in 48-hour group in this study, and in soman study (11), suggesting that a high dose of OP that induces seizures is required for this type of gut dysbiosis.

Importantly, with the exception of a few genera, there were minimal taxonomic changes 5 weeks post-DFP intoxication similar to the soman study which reported that taxonomic changes were negligible 75 days post-exposure (Getnet et al., 2018). As we have shown in previous studies, DFP-induced SE leads to the development of spontaneous recurrent seizures in most of the animals (Puttachary et al., 2016; Putra et al., 2020a). Although we did not utilize telemetry devices in these animals to monitor seizures, it is likely that these animals may have been experiencing spontaneous seizures by 5 weeks. Thus, our data would suggest that gut dysbiosis is not contributing to the initiation of seizures during an epileptic phase. In contrast, a study in children with epilepsy found a unique microbial signature that might contribute to drug resistance which might suggest that the microbiome component may be model dependent (Ceccarani et al., 2021). Future studies will further investigate the relationship between gut dysbiosis and spontaneous recurrent seizures in this rodent model.

We also found changes in microbial diversity at 48 hours and 7 days post intoxication which did not persist at 5 weeks. Loss of microbial diversity is generally considered to be indicative of compromised health (Mosca et al., 2016; Eisenstein, 2020). Alpha diversity measures intra-sample diversity while beta diversity measures inter-sample diversity. In our study, DFP treated animals had reduced alpha diversity at 48 hours using the observed, chao1, ACE and Fisher metrics of which the first three only take abundance into account which would suggest that the alpha diversity changes are primarily concerned with abundance rather than evenness (Willis, 2019). Fisher’s alpha diversity takes into account the number of species as well as the number of individuals in those species (Fisher et al., 1943). Although there were no statistical differences in alpha diversity at later timepoints, it did appear that the change in Beta diversity persisted at 7 days but not at 5 weeks which agrees with the significant changes we observed at the phylogenetic level.

Although it is important to understand the impact of acute OP nerve agent toxicity, our study also examined the impact of an orally active disease modifier, SAR, in mediating gut dysbiosis. We thought that the changes in the gut microbiome could influence the metabolism or efficacy of any orally administered disease modifier. It is known that SAR is bioactivated by cytochrome P450, a drug metabolizing enzymes that is known to be expressed in some bacteria (Murphy, 2015; Chen et al., 2016). SAR is a potent inhibitor of src family kinases which have been implicated in various neurological diseases such as Alzheimer’s disease, Parkinson’s disease, and epilepsy (Green et al., 2009; Nygaard, 2018; Panicker et al., 2019; Putra et al., 2020b; Sharma et al., 2021). We have previously tested SAR in the DFP model and found that, depending on the initial SE severity, early administration can mitigate the epileptogenic markers such as seizures, neuroinflammation and neurodegeneration (Gage et al., 2021a; Gage et al., 2021b). Although we did not quantify, in our previous experiments we occasionally observed increased prevalence of diarrhea in the SAR treated animals with higher doses that were also challenged with DFP which led to some concerns on how higher doses of SAR might have influenced the gut microbiome. Importantly, in this study we did not observe many changes in gut microbiome between the vehicle and the SAR treated groups suggesting that SAR at optimal dose *via* oral route is safe.

In this study, we were able to successfully determine the impact of DFP and SAR on gut dysbiosis. Future studies could further address dysbiosis in this model by considering other factors such as sex and age as both are well known to impact the gut microbiome (Kim et al., 2020; Bosco and Noti, 2021). Also of interest, it is also well known that prolonged seizures, induced by DFP and other chemoconvulsants, impact both cognitive function and motor ability (Hernandez et al., 2002; Holmes, 2015; Helmstaedter and Witt, 2017; Guignet et al., 2019; Putra et al., 2020b). As cognition and motor ability are also associated with the gut microbiome (Sampson et al., 2016; Meyer et al., 2022), it would be interesting to explore their relationship in the DFP model. However, in the DFP model, animals were morbid (required 5-7 days to recover their bodyweight, some animals had spontaneous seizures while handling) (Gage et al., 2021b). Therefore, conducting behavioral tests to determine the early effects of gut dysbiosis was not feasible in this study. However, it is possible that the short-term dysbiosis observed in this study could contribute to long-term neurobehavioral deficits. Changes in metabolites are also common to both gut dysbiosis and chemoconvulsant-induced epilepsy (Meldrum and Chapman, 1999; Agus et al., 2021). For example, in a recent study, we observed an increased concentration of cresols and alterations in dopaminergic neurotransmission within two days of induction of gut dysbiosis and the resultant secondary proliferation of major cresol-producing bacteria (Vinithakumari et al., 2022). In addition, acute gut permeability changes could increase serum toxic metabolite concentrations (Chakaroun et al., 2020; Ghosh et al., 2020; Ma et al., 2022). It would be interesting to investigate in future studies whether gut-induced changes in critical metabolites contribute to the toxicity caused by OPNAs. It is also important to consider whether the findings in this study are translatable to humans. Although there is no data on the microbiome of humans exposed to OPNAs, pesticides have been implicated in altering the human gut microbiome, mostly using *in vitro* studies (Utembe and Kamng’ona, 2021). It is likely that our findings in rats exposed to DFP might translate to humans exposed to OPNAs.

## 5 Conclusion

The purpose of the current study was to determine the overall impact of the OP, DFP and the disease modifier, SAR on the gut microbiome. We found that DFP-induced dysbiosis at 48 hours and 7 days were no longer prevalent at 5 weeks post intoxication. We also observed expected changes in alpha and beta diversities. Interestingly, SAR did not affect DFP-induced gut microbiome changes which are required to metabolize DFP. Importantly the function of many of these gut-microbiota is diverse and there are several unknowns about the role of each bacterium in OP-induced gut motility, localized gut immunity, and absorption. As research progresses in the gut microbiome field, we might better understand how these microorganisms are contributing to health and epileptogenesis following OP intoxication. Targeting changes in the gut microbiome at an appropriate time might be a future therapeutic approach to improve the efficacy of orally acting drugs.

## Supplementary Material

Supplementary Figures S1-S7

Table S1

## Data Availability

The datasets presented in this study can be found in online repositories. The names of the repository/repositories and accession number(s) can be found below: BioProject, accession number PRJNA865374.
