# Supplementary material for: Gut dysbiosis following organophosphate, diisopropylfluorophosphate (DFP), intoxication and saracatinib oral administration
Source: Front Microbiomes. 2022 Oct 20;1:1006078. doi: 10.3389/frmbi.2022.1006078 (PMC10256240; doi:10.3389/frmbi.2022.1006078)
Supplement: Supplementary file 1 [file Table_1.docx]

| Time point | Analysis type | F-value | R^2^ | P |
| --- | --- | --- | --- | --- |
| 48 hours | PCoA | 3.1218 | 0.40082 | 0.002 |
| 48 hours | NMDS | 3.1218 | 0.40082 | 0.002 |
| 7 days | PCoA | 3.3408 | 0.38514 | 0.001 |
| 7 days | NMDS | 3.3408 | 0.38514 | 0.001 |
| 5 weeks | PCoA | 1.8274 | 0.2552 | 0.012 |
| 5 weeks | NMDS | 1.8274 | 0.2552 | 0.011 |

Table S1. PERMANOVA values for measuring Beta diversity
