# Supplementary material for: Gut dysbiosis following organophosphate, diisopropylfluorophosphate (DFP), intoxication and saracatinib oral administration
Source: Front Microbiomes. 2022 Oct 20;1:1006078. doi: 10.3389/frmbi.2022.1006078 (PMC10256240; doi:10.3389/frmbi.2022.1006078)
Supplement: Supplementary file 2 [file DataSheet_1.pdf]

Supplemental figures

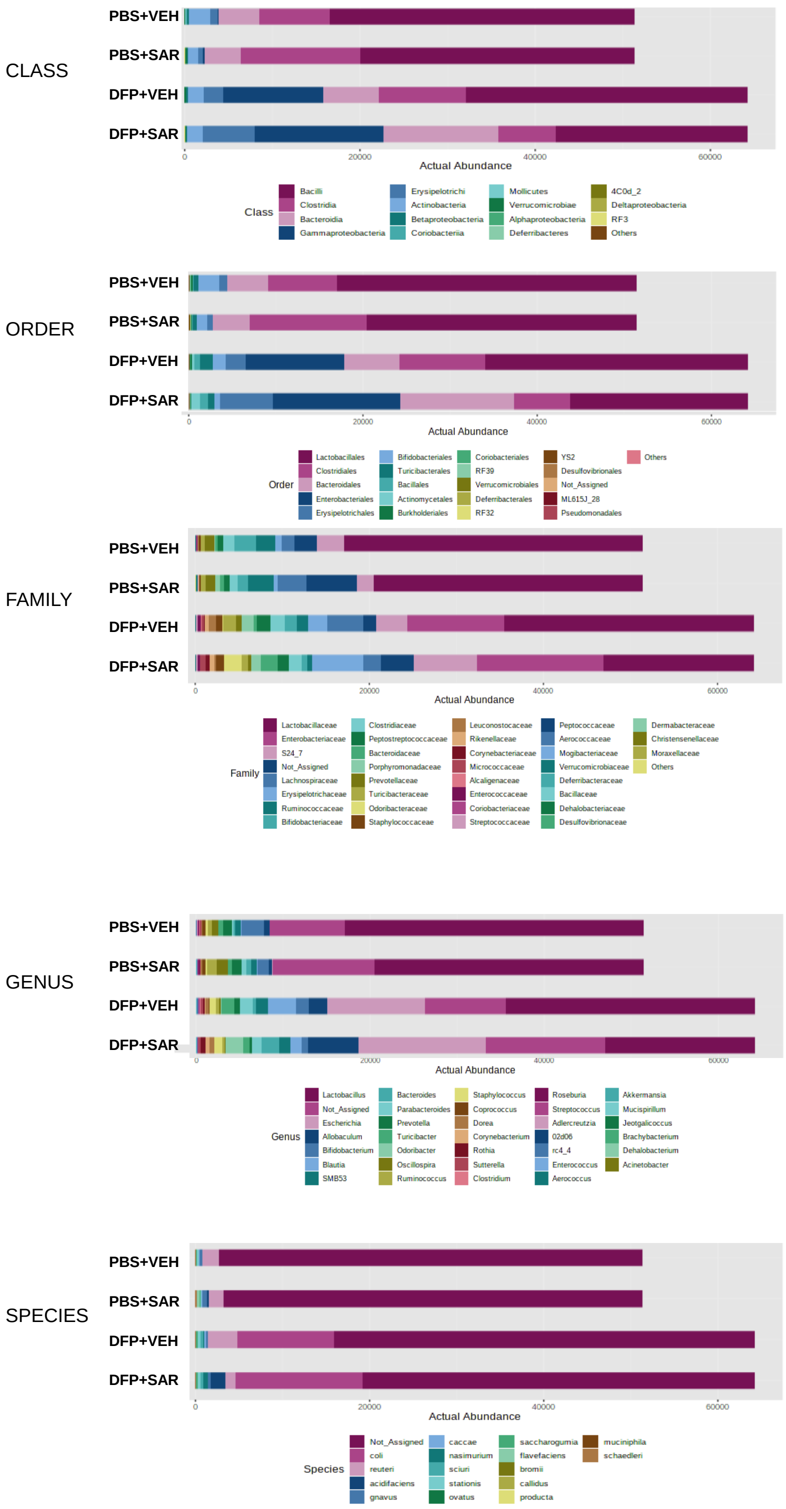

Figure S1. Impact of DFP and SAR on taxonomic levels at 48 hours post-exposure.

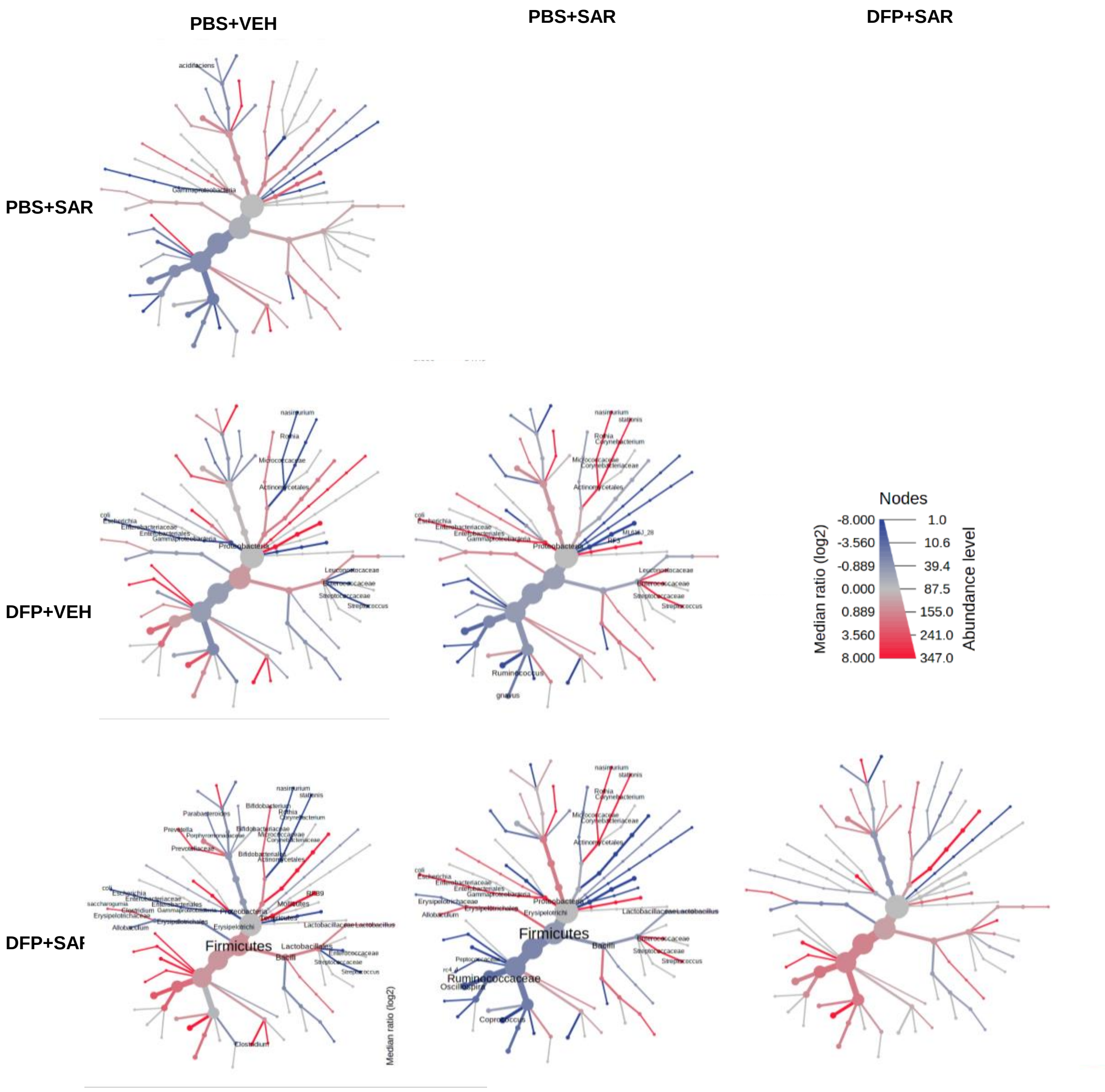

Figure S2. Heat tree comparing treatment groups at the species level at 48 hours post-exposure.

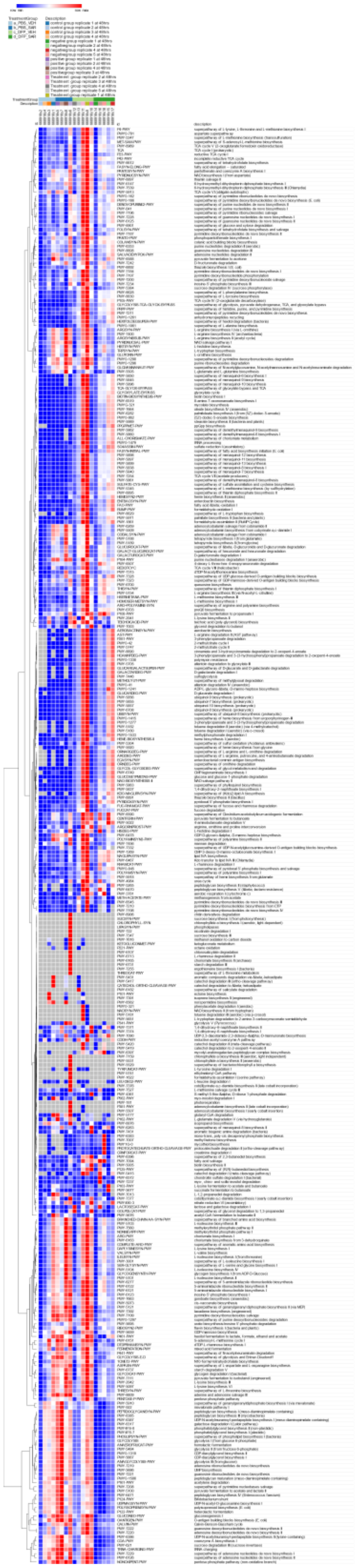

Figure S3. Functional composition predictions at 48 hours post-exposure.

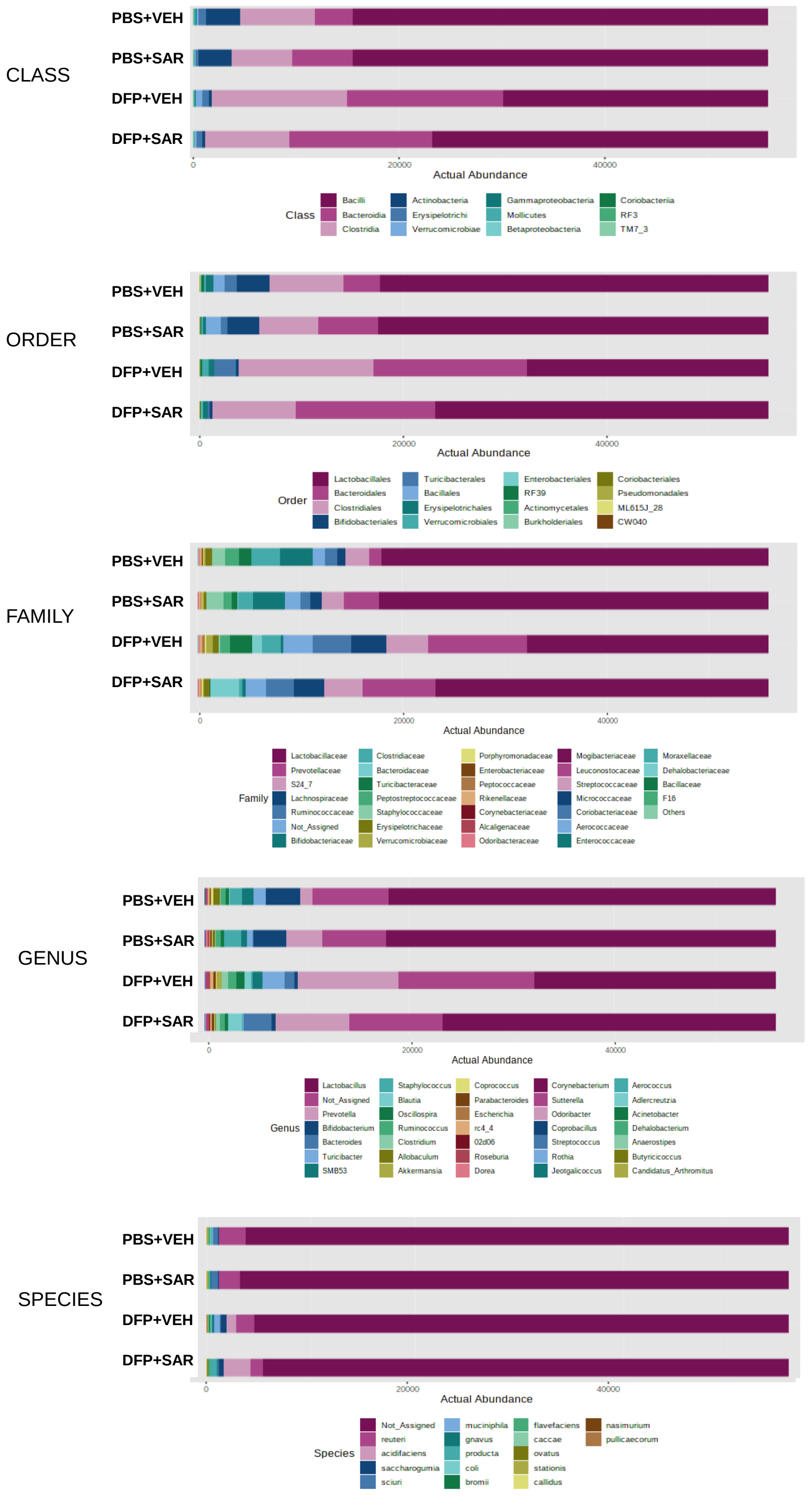

Figure S4. Impact of DFP and SAR on taxonomic levels at 7 days post-exposure.

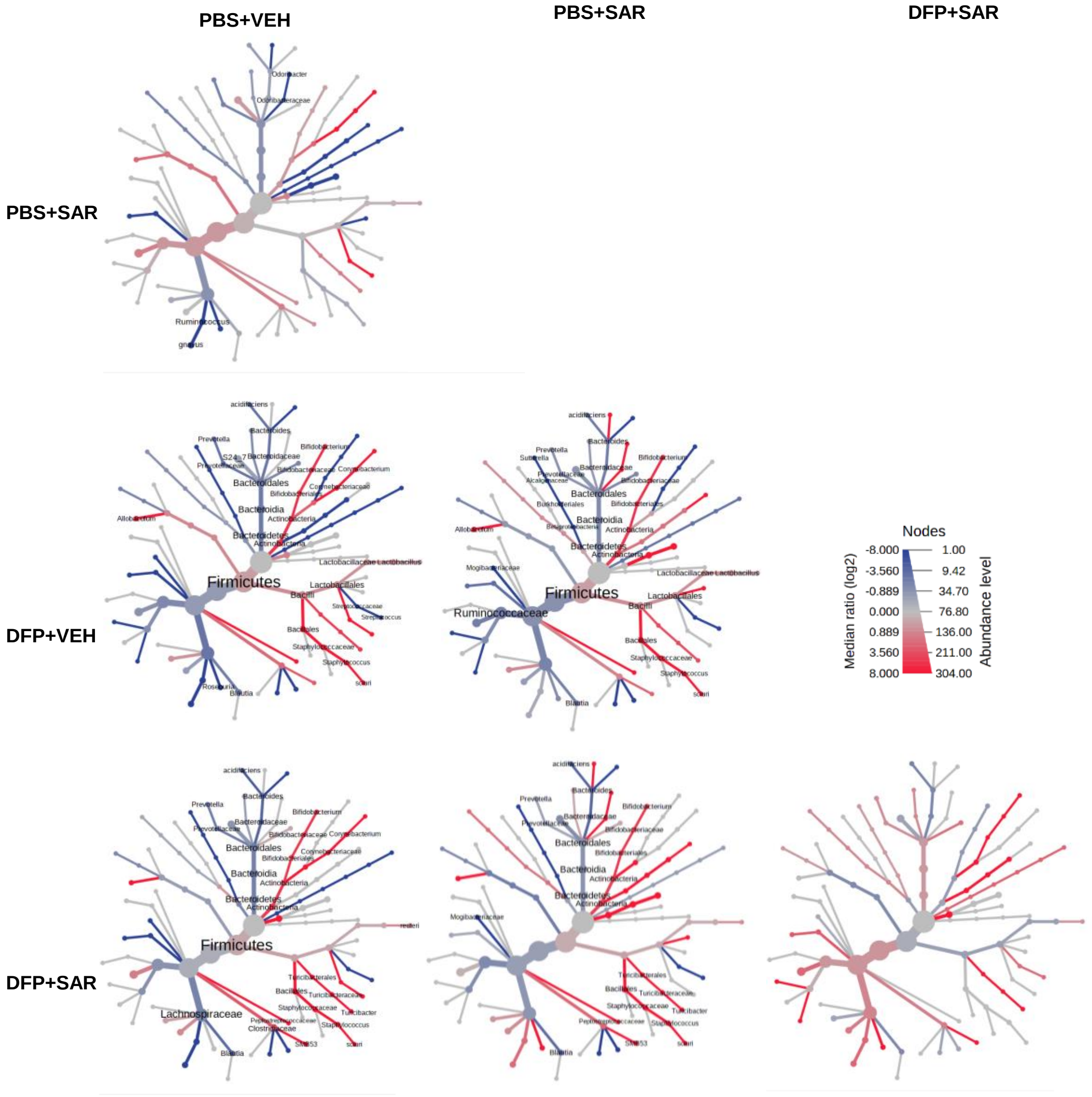

Figure S5. Heat tree comparing treatment groups at the species level at 7 days post-exposure.

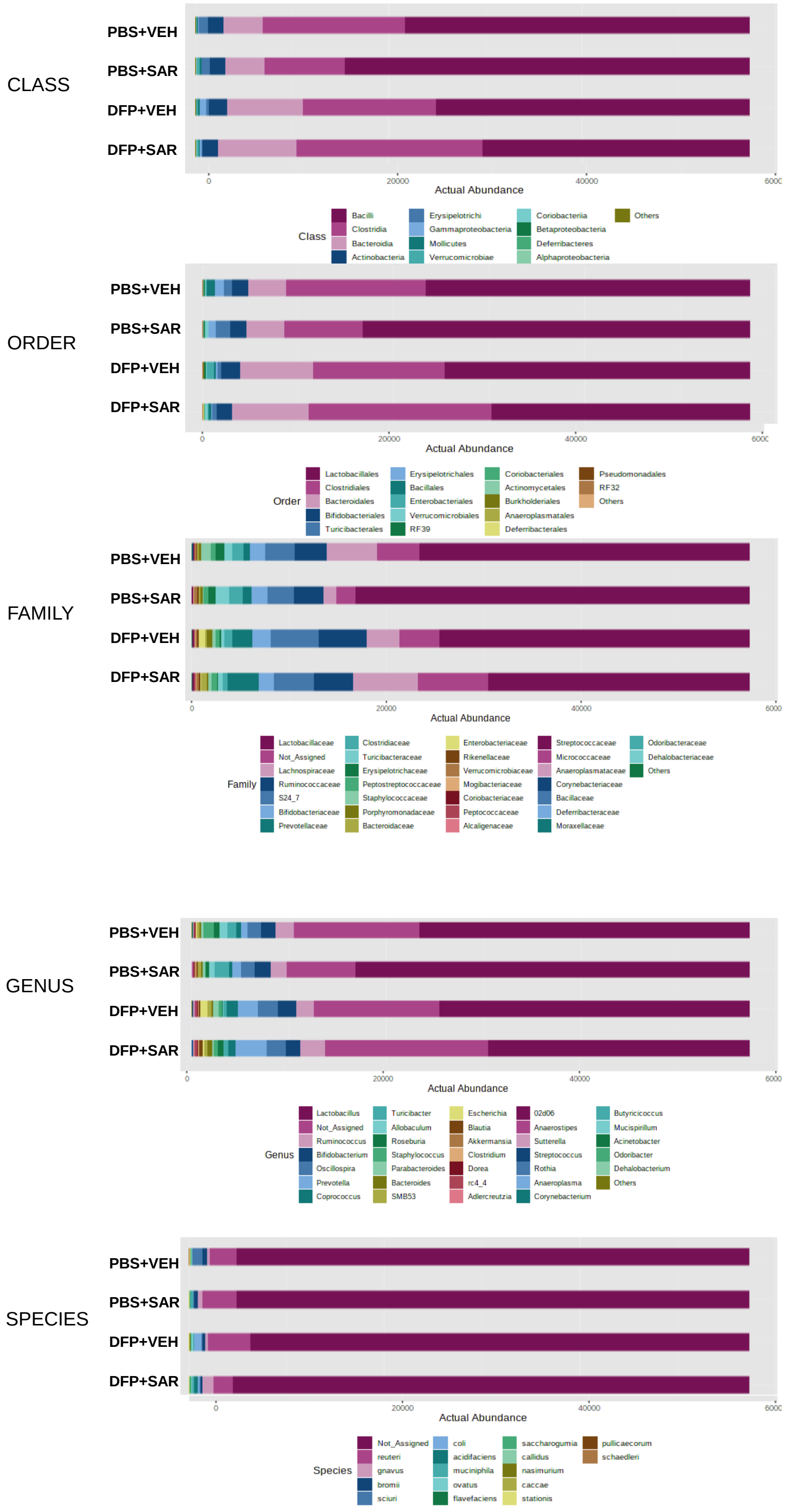

Figure S6. Impact of DFP and SAR on taxonomic levels at 5 weeks post-exposure.

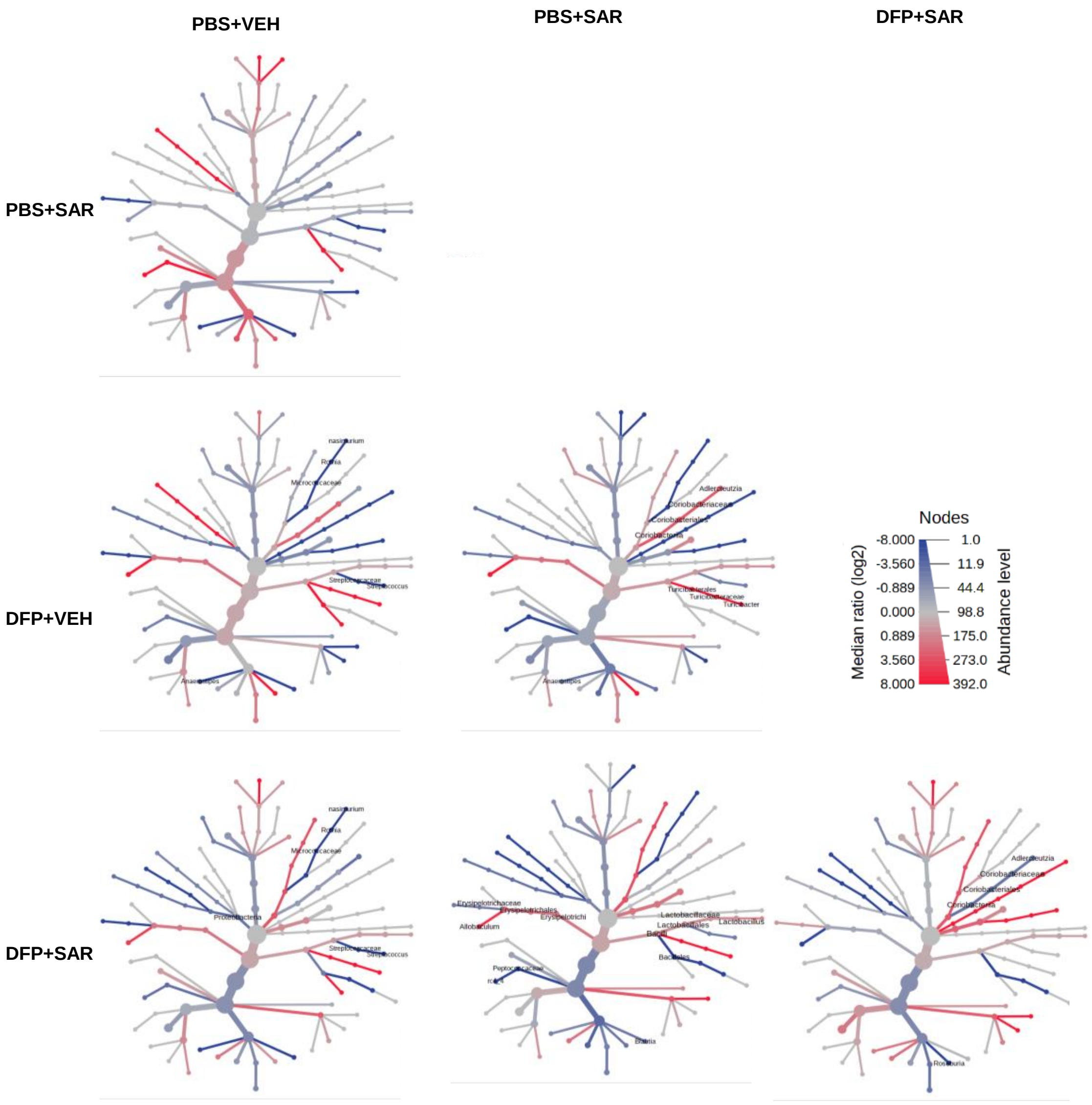

Figure S7. Heat tree comparing treatment groups at the species level at 5 weeks post-exposure.
