# Supplementary material for: Gut dysbiosis following organophosphate, diisopropylfluorophosphate (DFP), intoxication and saracatinib oral administration
Source: Front Microbiomes. Author manuscript; Available in PMC 2023 Jun 9. (PMC10256240; doi:10.3389/frmbi.2022.1006078)
Supplement: Supplementary file 1 [file NIHMS1898588-supplement-Supplementary_Figures_S1-S7.pdf]

# Supplemental figures

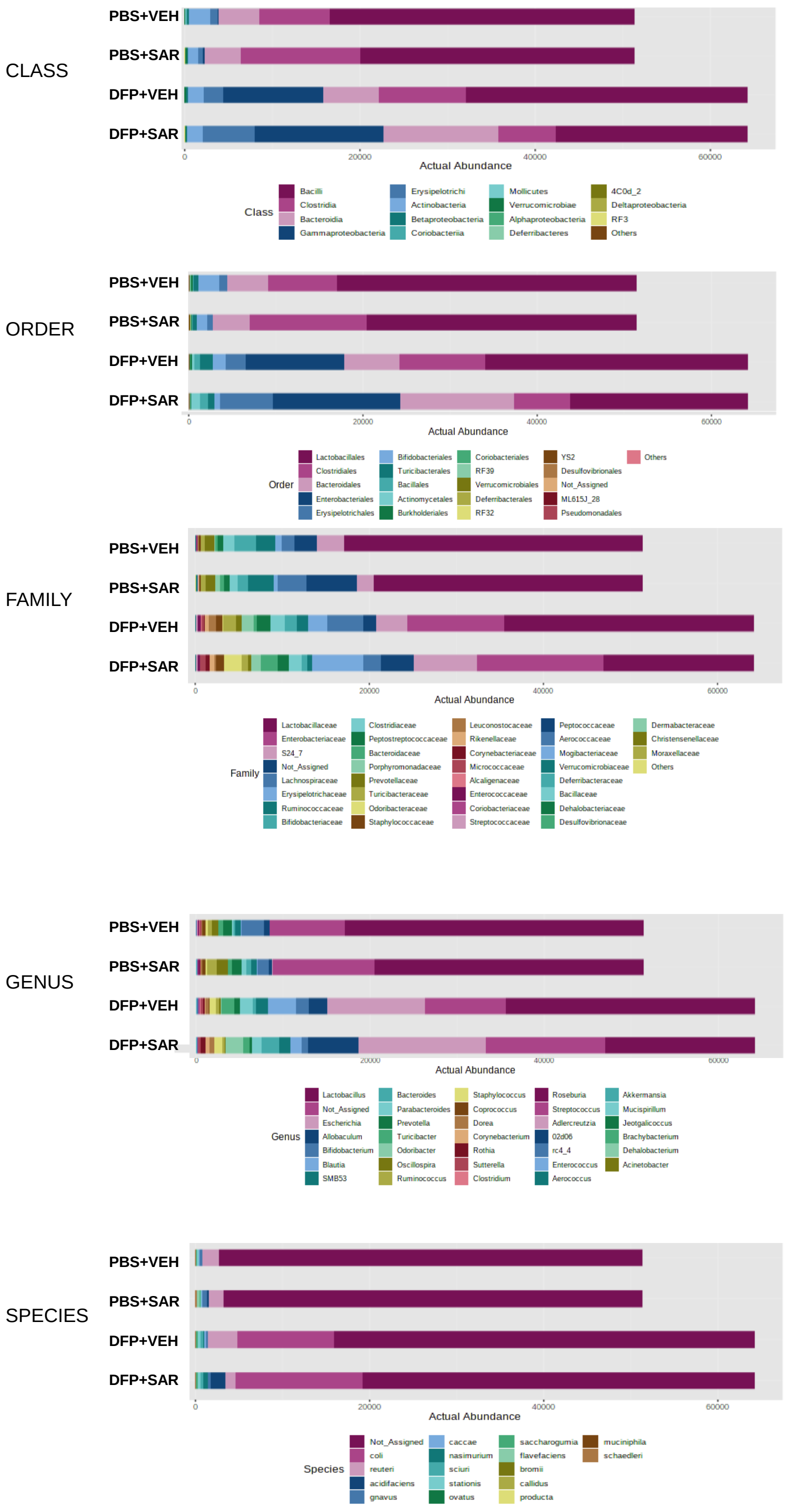


**Figure S1**. Impact of DFP and SAR on taxonomic levels at 48 hours post-exposure.

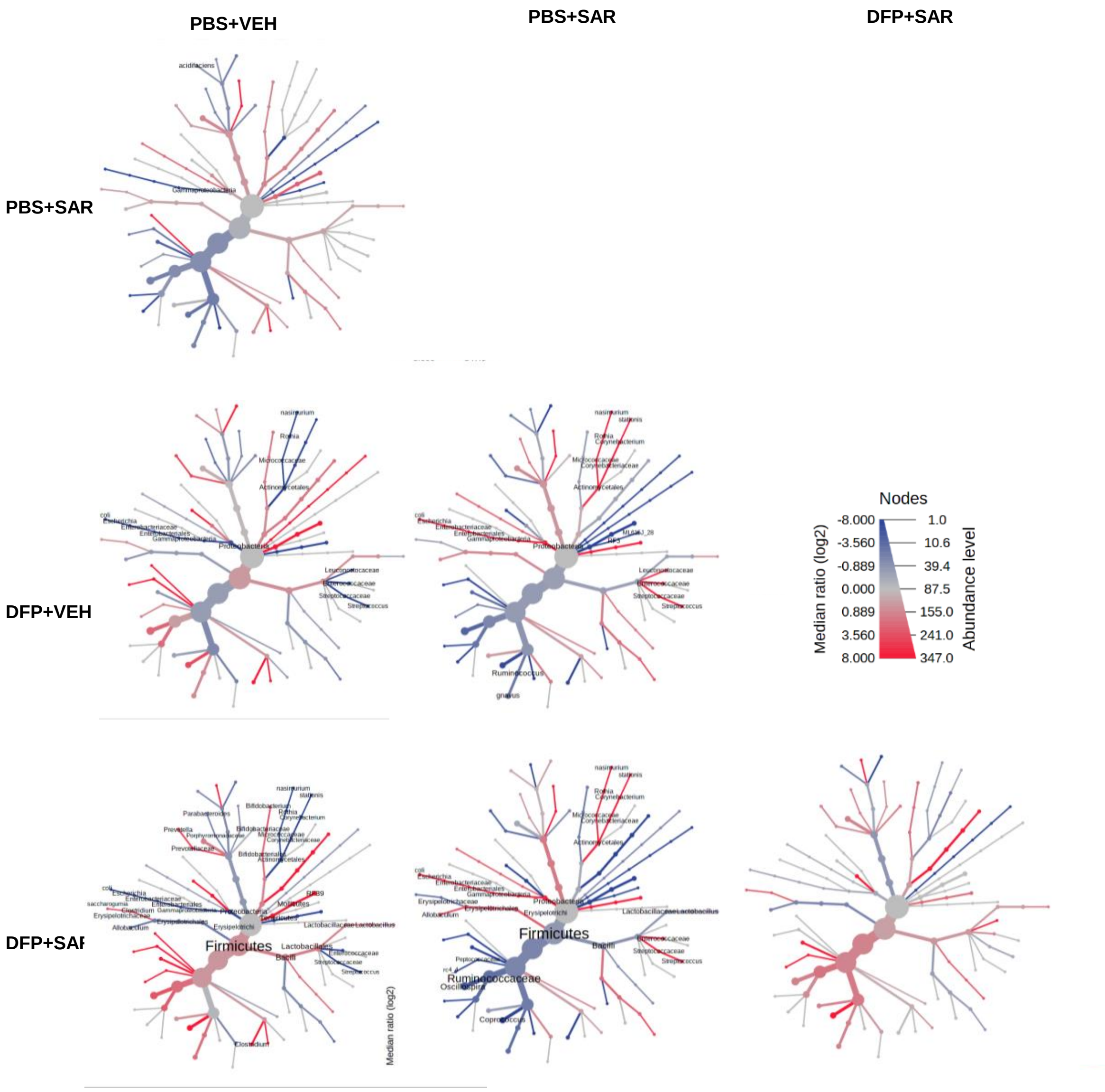


**Figure S2**. Heat tree comparing treatment groups at the species level at 48 hours post-exposure.

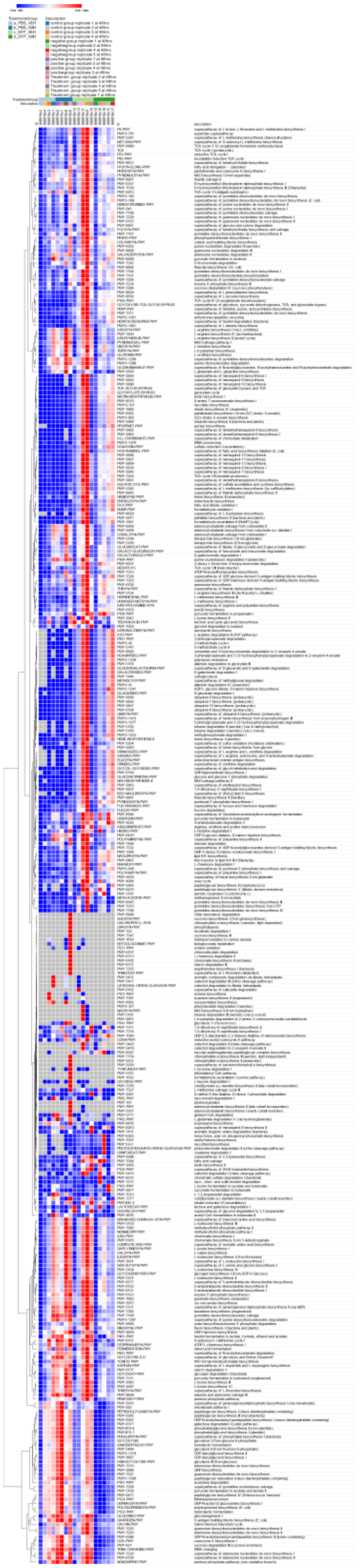

**Figure S3.** Functional composition predictions at 48 hours post-exposure.

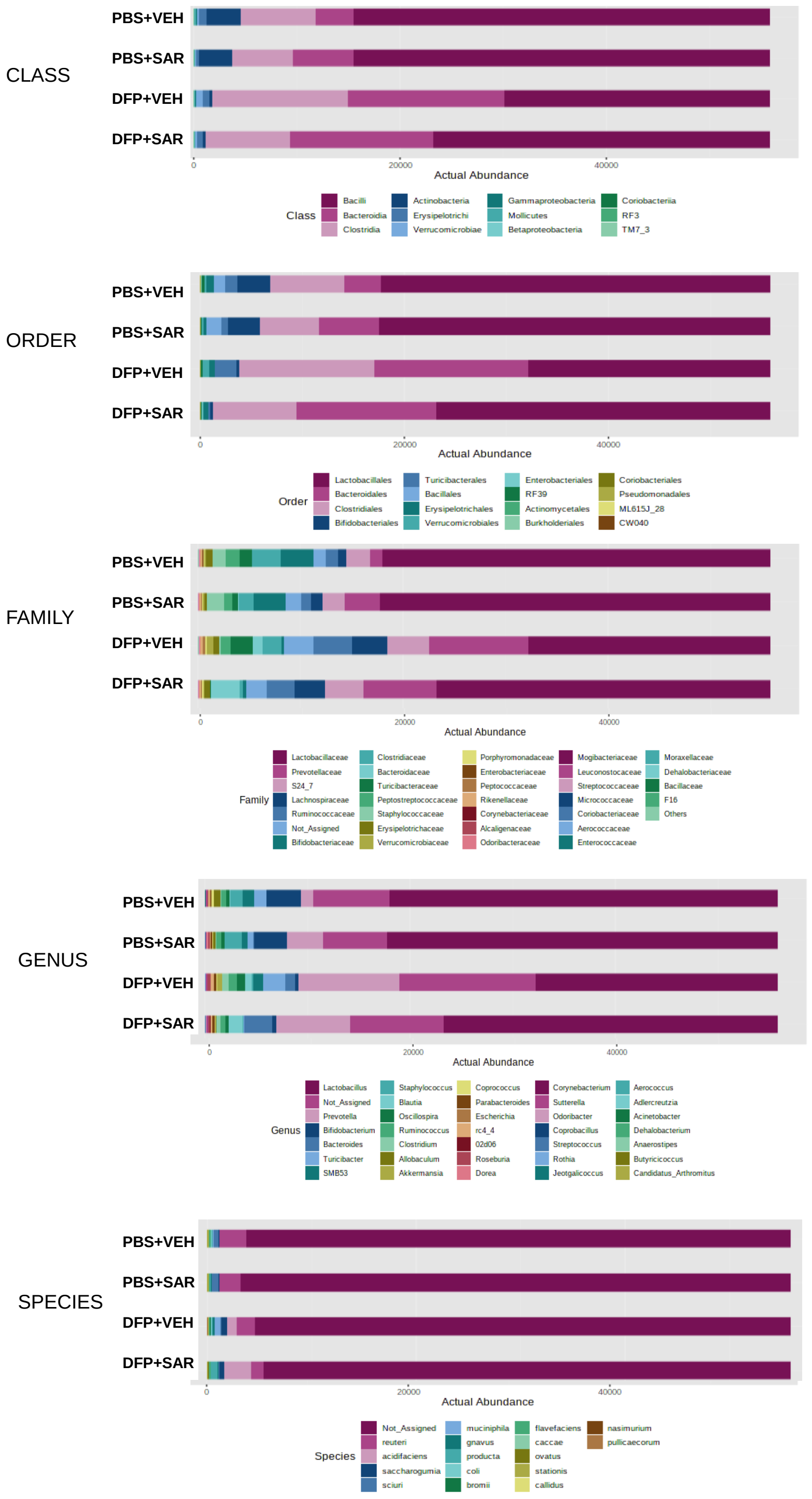


**Figure S4**. Impact of DFP and SAR on taxonomic levels at 7 days post-exposure.

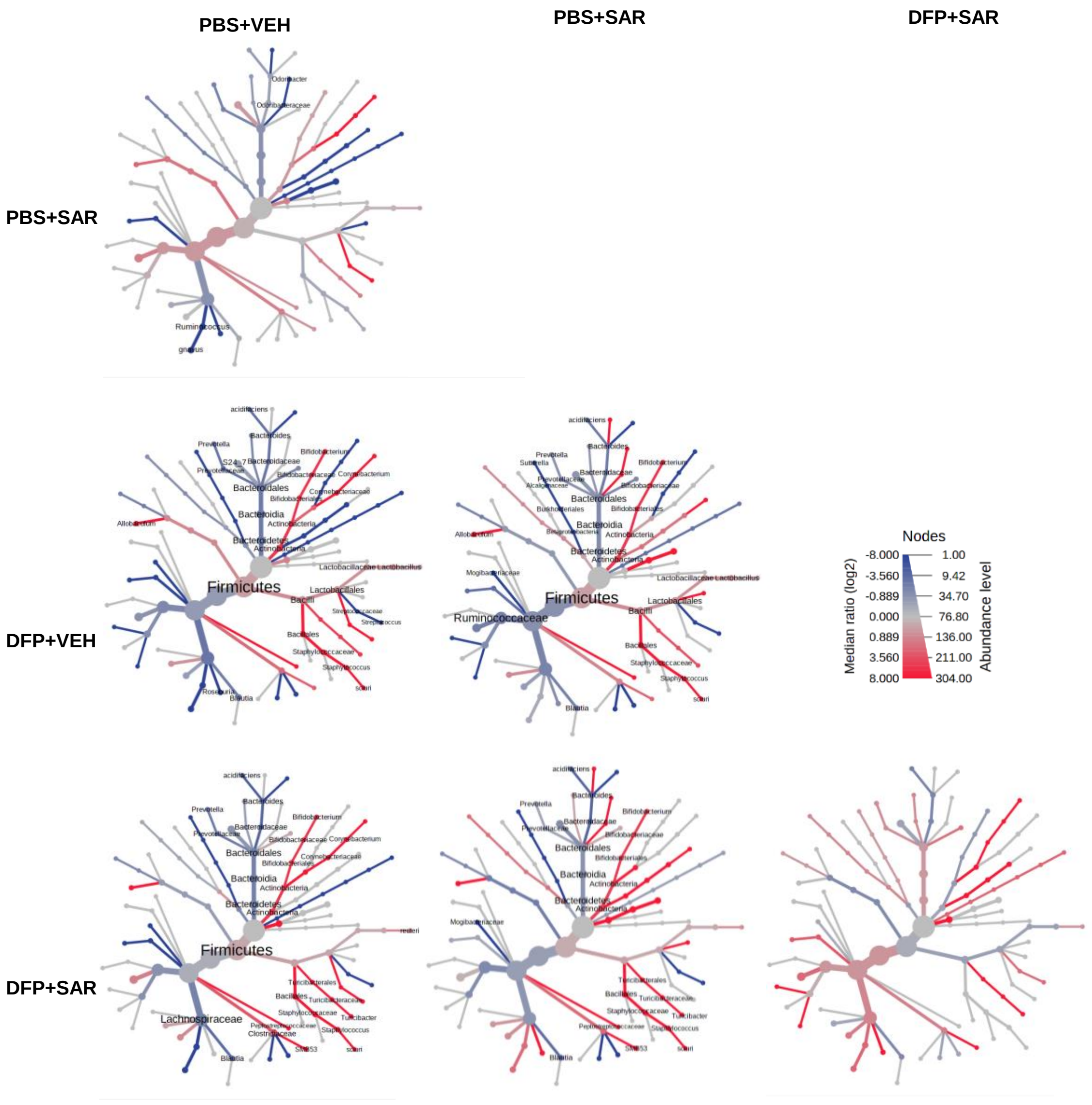


**Figure S5**. Heat tree comparing treatment groups at the species level at 7 days post-exposure.

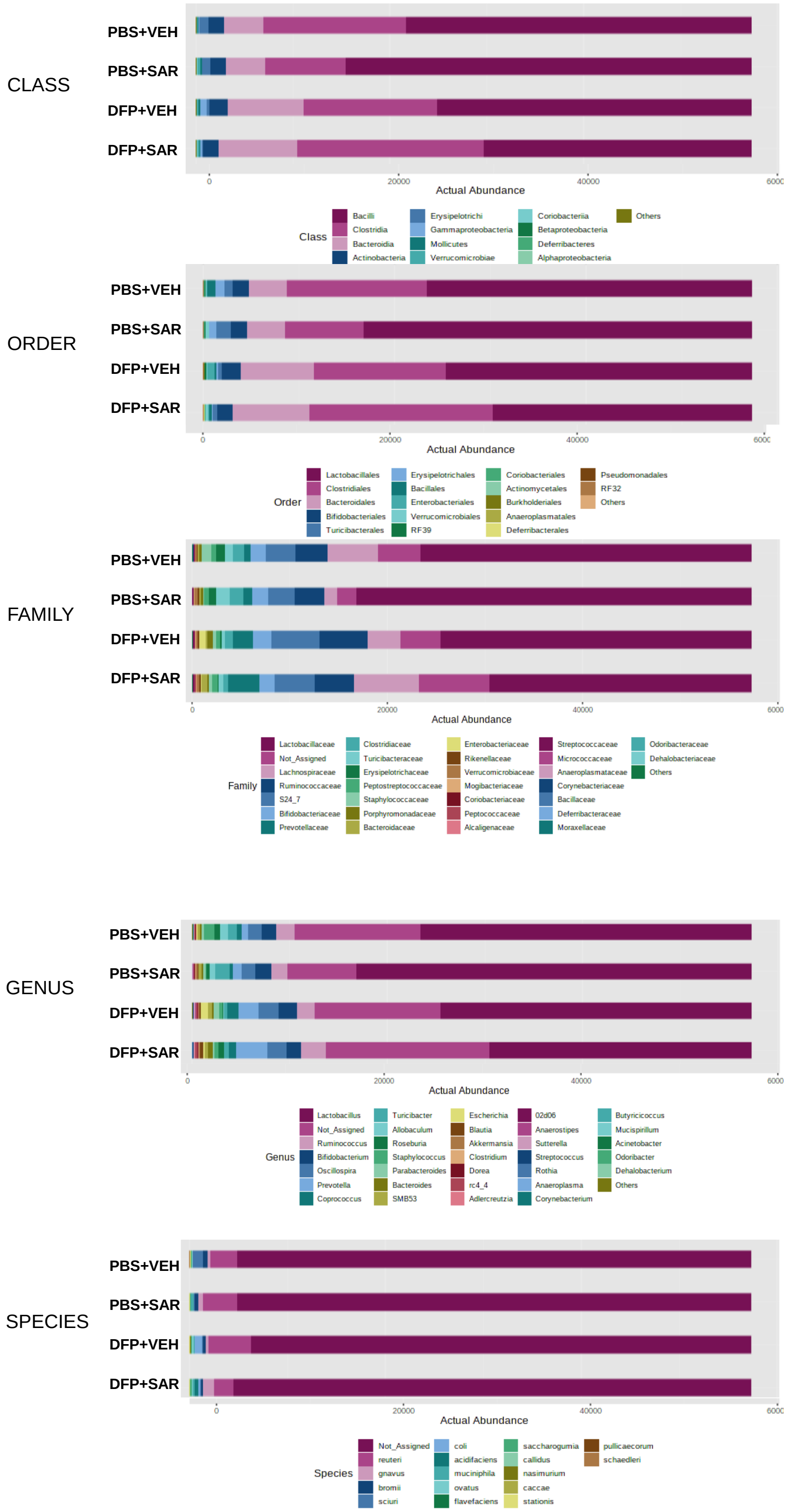


**Figure S6**. Impact of DFP and SAR on taxonomic levels at 5 weeks post-exposure.

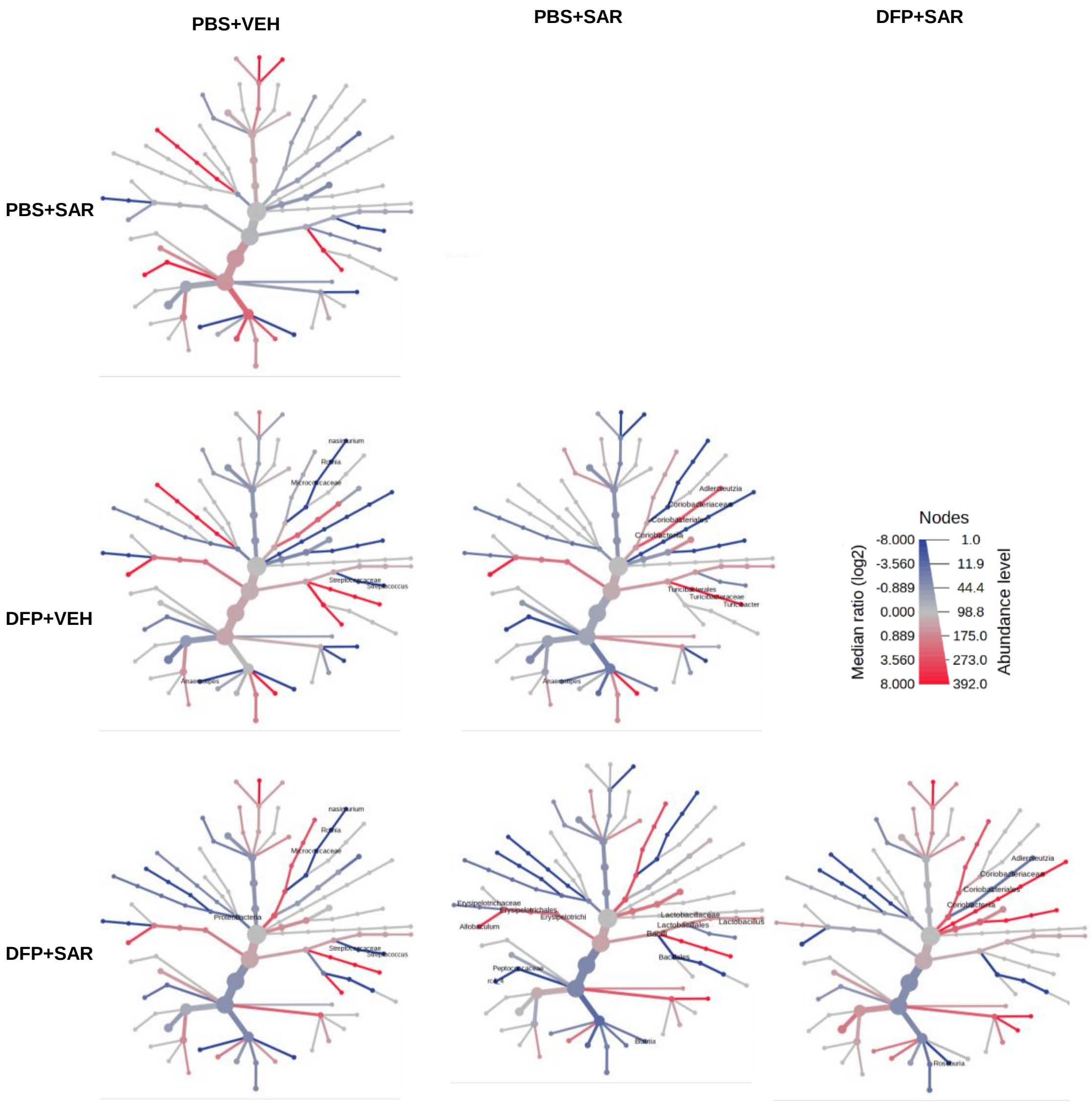


**Figure S7**. Heat tree comparing treatment groups at the species level at 5 weeks post-exposure.
